# Exploring the shared mechanism of fatigue between systemic lupus erythematosus and myalgic encephalomyelitis/chronic fatigue syndrome: monocytic dysregulation and drug repurposing

**DOI:** 10.3389/fimmu.2024.1440922

**Published:** 2025-01-07

**Authors:** Daisi Zheng, Xiaolong Li, Peicheng Wang, Qingmiao Zhu, Zhiyan Huang, Ting Zhao

**Affiliations:** ^1^ The First Affiliated Hospital of Zhejiang Chinese Medical University, Hangzhou, China; ^2^ College of Basic Medical Sciences, Zhejiang Chinese Medical University, Hangzhou, China; ^3^ Key Laboratory of Chinese Medicine Rheumatology of Zhejiang Province, Research Institute of Chinese Medical Clinical Foundation and Immunology, College of Basic Medical Science, Zhejiang Chinese Medical University, Hangzhou, China

**Keywords:** systemic lupus erythematosus, myalgic encephalomyelitis/chronic fatigue syndrome, fatigue, bioinformatics analysis, single-cell transcriptomics

## Abstract

**Background:**

SLE and ME/CFS both present significant fatigue and share immune dysregulation. The mechanisms underlying fatigue in these disorders remain unclear, and there are no standardized treatments. This study aims to explore shared mechanisms and predict potential therapeutic drugs for fatigue in SLE and ME/CFS.

**Methods:**

Genes associated with SLE and ME/CFS were collected from disease target and clinical sample databases to identify overlapping genes. Bioinformatics analyses, including GO, KEGG, PPI network construction, and key target identification, were performed. ROC curve and correlation analysis of key targets, along with single-cell clustering, were conducted to validate their expression in different cell types. Additionally, an inflammation model was established using THP-1 cells to simulate monocyte activation in both diseases *in vitro*, and RT-qPCR was used to validate the expression of the key targets. A TF-mRNA-miRNA co-regulatory network was constructed, followed by drug prediction and molecular docking.

**Results:**

Fifty-eight overlapping genes were identified, mainly involved in innate immunity and inflammation. Five key targets were identified (IL1β, CCL2, TLR2, STAT1, IFIH1). Single-cell sequencing revealed that monocytes are enriched with these targets. RT-qPCR confirmed significant upregulation of these targets in the model group. A co-regulatory network was constructed, and ten potential drugs, including suloctidil, N-Acetyl-L-cysteine, simvastatin, ACMC-20mvek, and camptothecin, were predicted. Simvastatin and camptothecin showed high affinity for the key targets.

**Conclusion:**

SLE and ME/CFS share immune and inflammatory pathways. The identified key targets are predominantly enriched in monocytes at the single-cell level, suggesting that classical monocytes may be crucial in linking inflammation and fatigue. RT-qPCR confirmed upregulation in activated monocytes. The TF-mRNA-miRNA network provides a foundation for future research, and drug prediction suggests N-Acetyl-L-cysteine and camptothecin as potential therapies.

## 1 Introduction

Systemic lupus erythematosus (SLE) is an autoimmune disease with an unclear pathogenesis. The breakdown of immune tolerance, characterized by abnormal T and B cell function, contributes to the production and deposition of autoantibodies, leading to multisystem damage (1). Fatigue is considered one of the most common symptoms in the onset and progression of SLE. It is reported by 67%-90% of patients and substantially affects their quality of life (2–4). It is noteworthy that patients still have significant fatigue even when clinical symptoms and serological markers are well-controlled (5–7). While several factors contributing to fatigue in SLE have been proposed, the precise etiology and mechanisms remain unclear in most patients, and effective treatment is lacking (4). Thus, investigating the mechanisms of fatigue in SLE is essential and valuable. It may aid in finding therapeutic approaches and drugs to reduce fatigue.

Myalgic encephalomyelitis/chronic fatigue syndrome (ME/CFS) is a diagnosis of exclusion, primarily characterized by severe and unrelenting fatigue lasting more than six months (8, 9). The pathogenesis of ME/CFS is also unclear, but etiological exploration suggests that it may be related to genetic predisposition, viral infections, and abnormal immune responses (10). ME/CFS shares certain similarities with SLE, such as fatigue, swollen lymph nodes, musculoskeletal pain, and cognitive dysfunction (11–14). Infections such as Epstein-Barr virus, Parvovirus B19, and Human Herpesvirus establish an etiological connection between SLE and ME/CFS (10, 15, 16). As understanding deepens, more details about immune dysfunctions are gradually revealed. The tyrosine phosphatase non-receptor type 22 (*PTPN22*) SNP rs2476601 and the cytotoxic T-lymphocyte-associated protein 4 (*CTLA4*) SNP rs3087243 have also been shown to be associated with the risk of developing SLE and ME/CFS (17). A subset of ME/CFS patients have elevated immunoglobulin levels, and even the presence of antinuclear antibodies and anti-dsDNA, which are consistent with SLE patients (18). In ME/CFS patients, similar cellular characteristics can be observed as in SLE patients, such as the dysregulation of T cells, B cells, and monocytes (9, 19, 20). These insights suggest a more intricate link in the pathogenesis of both SLE and ME/CFS, beyond the common symptom of fatigue. Therefore, we hypothesize that there are common pathways and immune cell dysregulation between SLE and ME/CFS, leading to their similar symptoms.

**Figure 1:**
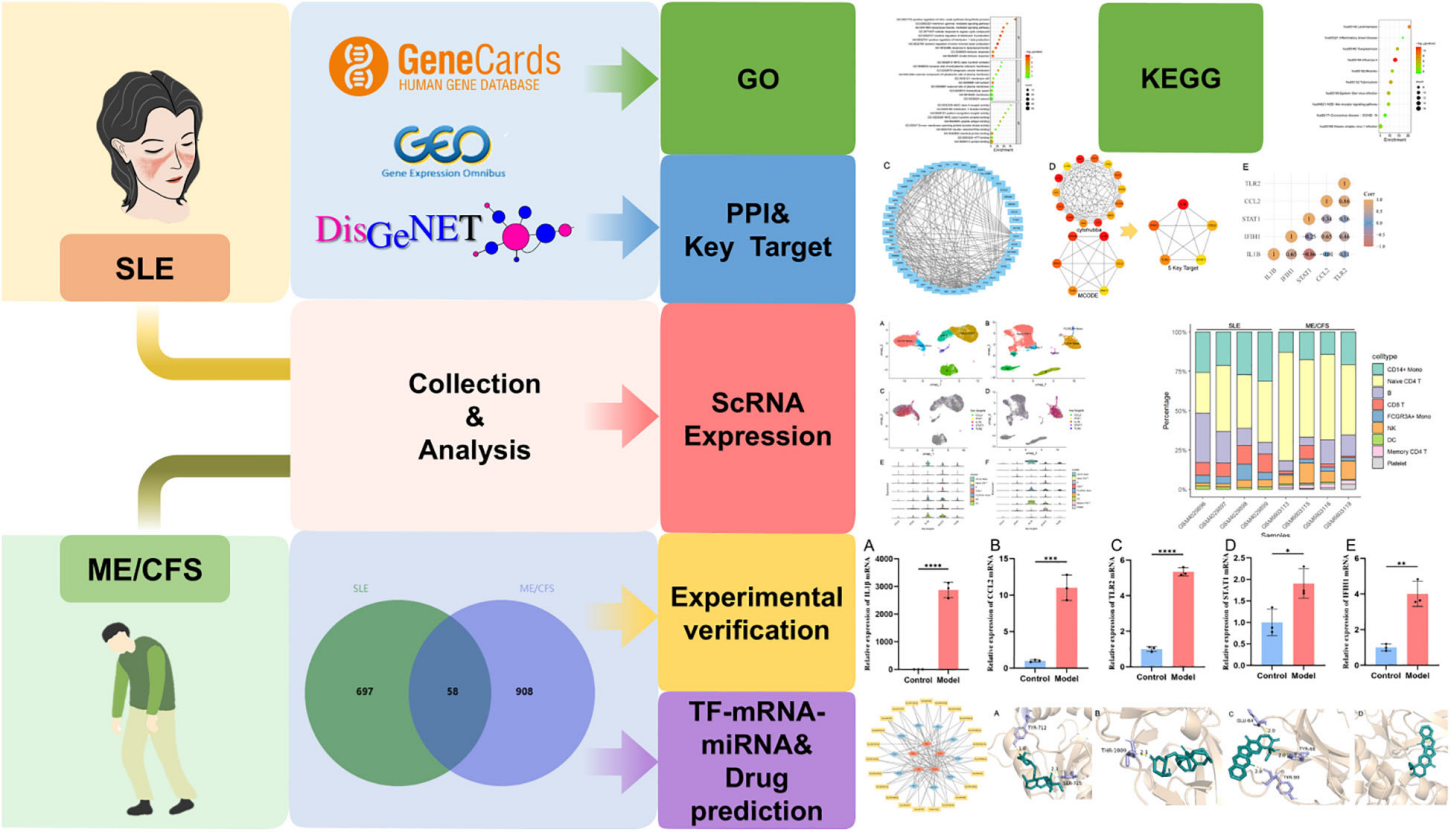
Workflow of our research.

Bioinformatics methods are essential for the discovery of unknown targets and mechanisms of disease. The overlapping genes could offer valuable insights into fatigue associated with SLE and ME/CFS. This study applied bioinformatics approaches including analysis of gene ontology (GO), Kyoto Encyclopedia of Genes and Genomes (KEGG), protein-protein interaction (PPI), single-cell transcriptomics and TF-mRNA-miRNA co-regulatory network. Furthermore, we predicted potential drugs using the DSigDB database and measured drug molecule affinity through molecular docking. **Figure 1** illustrates our research workflow.

## 2 Materials and methods

### 2.1 Collecting overlapping genes between SLE and ME/CFS

The Gene Expression Omnibus (GEO) (http://www.ncbi.nlm.nih.gov/geo/) is a database that contains a wide range of disease-related datasets from high-throughput sequencing and microarray studies. For SLE, we selected the dataset GSE72326 - GPL10558, comprising 157 SLE samples and 20 healthy controls. This dataset was chosen for its peripheral blood analysis and large sample size, which enhances statistical robustness. For ME/CFS, we selected the dataset GSE14577 - GPL96, consisting of 8 ME/CFS samples and 7 healthy controls. GSE14577 was included because it was the only GEO dataset that specifically analyzed peripheral blood in ME/CFS patients, providing unique insights despite its smaller sample size. The limma package is based on a linear model and uses weighted least squares to estimate gene expression differences, while employing a Bayesian method to adjust for multiple testing issues. The datasets GSE72326 and GSE14577 were converted into expression matrices and categorized, and analyzed using the limma package (version: 3.40.2) in R software (v4.0.3). We examined the adjusted *p*-values (adj. *p*Value) to correct for false positives. We defined the threshold for filtering differentially expressed genes (DEGs) as an adj. *p*Value < 0.05 and | fold change (FC) | ≥ 1.3. To supplement the smaller number of DEGs obtained for ME/CFS, we selected relevant genes from the DisGeNet (https://disgenet.com/) (21) and GeneCards (https://www.genecards.org/) (22) databases. GeneCards provides disease-related gene scores through its built-in mining algorithms, and we selected targets with a relevance score > 25 as high-confidence targets for ME/CFS. Given the relatively small variance in scoring and the limited number of genes from DisGeNet, we included all associated genes from that database. Subsequently, we removed duplicate entries from the gene lists obtained from the three databases. An online Venn diagram tool (https://www.bioinformatics.com.cn/static/others/jvenn/example.html) was used to identify the overlapping genes between SLE and ME/CFS.

### 2.2 Analysis of GO and KEGG

DAVID (23) (https://david.ncifcrf.gov/) is a comprehensive bioinformatics web tool used for gene functional annotation, gene set enrichment analysis, and data visualization. To identify the functions of the overlapping genes, we utilized the DAVID database to conduct GO and KEGG enrichment analyses. GO analysis encompasses three terms: biological processes (BP), cellular components (CC), and molecular functions (MF) (24). KEGG analysis aids in identifying the involvement of overlapping genes in signaling pathways. The enrichment analysis performed using the DAVID database evaluates gene enrichment in annotation terms through Fisher’s Exact Test. The false discovery rate (FDR) helps identify a high number of significant features while maintaining a relatively low proportion of false positives. We selected the top 10 terms with the lowest FDR from both the GO analysis (BP, CC, MF) and KEGG analysis as reliable enrichment results.

### 2.3 Obtaining key targets and performing correlation analysis

The construction of a PPI network helps to understand protein function and interaction mechanisms, and can be used to identify key targets. We used the STRING database (https://string-db.org) to generate a PPI network for the overlapping genes and visualized it using Cytoscape version 3.10.0. To obtain the key targets involved in fatigue associated with SLE and ME/CFS, we used both the cytoHubba plugin and the MCODE algorithm within Cytoscape. We used the MCC (Maximal Clique Centrality) algorithm in cytoHubba to extract the top 6 targets and identified the key targets module using MCODE. Proteins that are common between the results from cytoHubba and MCODE are considered key targets for SLE and ME/CFS. To delve deeper into the relationships among these key targets, correlation analysis was performed on SLE samples obtained from the GSE72326 dataset using the ggstatsplot package in R. Additionally, to assess the diagnostic potential of the identified key targets *(IL1β*, *CCL2*, *TLR2*, *STAT1*, and *IFIH1*), receiver operating characteristic (ROC) analysis was conducted. For this purpose, we utilized two independent datasets from the GEO database: GSE211700 (25), which includes SLE patient samples, and GSE128078 (26), which consists of ME/CFS patient samples. Receiver operating characteristic curve analysis was performed for key targets using the “rms” package, and the area under the curve (AUC) was selected to evaluate its diagnostic potential.

### 2.4 Single-cell clustering analysis

The scRNA-seq datasets GSE135779 (27) for SLE and GSE214284 (19) for ME/CFS were downloaded from GEO. Samples include GSM4029896, GSM4029897, GSM4029898, GSM4029899, GSM6603113, GSM6603115, GSM6603166 and GSM6603118. Downstream analysis was performed using the Seurat R package (version 4.4.1) (28). Data with fewer than 300 expressed genes or where genes were expressed in fewer than 5 cells were excluded. Violin plots depicting the proportions of ribosomal and mitochondrial genes were generated for quality control. The Harmony package was utilized to remove batch effects, and the FindVariableFeatures function was employed to identify highly variable genes. Dimensionality reduction and clustering were performed using ScaleData, RunPCA, RunTSNE, RunUMAP, FindNeighbors, and FindClusters. The resulting clusters were visualized using Uniform Manifold Approximation and Projection (UMAP). Classic marker genes were used to annotate subpopulations, and visualization was achieved using the ggplot2 package.

### 2.5 Validation of key target expression under monocyte activation

Based on the results from single-cell sequencing analysis, we preliminarily confirmed the accumulation of these key targets in monocytes. GO analysis also indicated a significant activation of the LPS pathway. To further validate the expression levels of these key targets in monocytes, we designed the following experiments. The model group consisted of THP-1 monocyte cells differentiated by PMA induction, followed by LPS incubation to induce an inflammatory phenotype. The control group consisted of untreated THP-1 monocytes. Quantitative Reverse Transcription Polymerase Chain Reaction (RT-qPCR) was then used to validate the expression of the five key targets in both the model and control groups. Statistical analysis was performed using an unpaired t-test.

#### 2.5.1 Cell culture and inflammatory model induction

THP-1 monocyte cells were cultured in RPMI-1640 medium supplemented with 10% fetal bovine serum (Gibco, 10099141C) and 1% penicillin-streptomycin (BL505A, Biosharp) in a 37°C, 5% CO2 incubator. For the model group, THP-1 cells were treated with 5 ng/mL phorbol 12-myristate 13-acetate (PMA, Sigma-Aldrich) for 48 hours to induce differentiation into adherent macrophages. Subsequently, cells were incubated with 100 μg/mL lipopolysaccharide (LPS, Sigma-Aldrich) for 24 hours to induce an inflammatory response (29). The control group consisted of untreated THP-1 cells.

### 2.5.2 Key target expression by RT-qPCR

Total RNA was extracted from the cells using the SteadyPure Fast RNA Extraction Kit (AG21023, ACCURATE BIOLOGY) according to the manufacturer’s protocol. The extracted RNA was then reverse transcribed into cDNA using the HiScript^®^ II Q RT Super Mix for qPCR (R223-01, Vazyme) following the manufacturer’s instructions. RT-qPCR was performed using the Pro Taq HS SYBR Green qPCR Kit (TaKaRa, RR820A), and gene expression was measured with the LightCycler 96 instrument (Roche, Basel, Switzerland). The cycle threshold (Ct) values of target genes were normalized to the β-actin housekeeping gene to obtain the ΔCt values. Relative gene expression levels were quantified using the 2-ΔΔCt method. The primer sequences for the housekeeping gene and the five key targets were designed by ACCURATE BIOLOGY (Hunan, China) and are listed in **Table 1**.

**Table 1:** Primer sequences for the housekeeping gene and five key targets.

Gene	Forward (5’-3’)	Reverse (5’-3’)
*β-actin*	TAGTCTCTCCCTCACGC CATCC	GTCACGCACGATTTCCCT CTCAG
*IL-1β*	CCAGGGACAGGATATG GAGCA	TTCAACACGCAGGACAGG TACAG
*CCL2*	CTCATAGCAGCCACCTTC ATTCC	AAGATCACAGCTTCTTTG GGACA
*TLR2*	ACCGTTTCCATGGC CTGTG	GATGTTCCTGCTGGGAG CTTTC
*STAT1*	TGTGCCAGCCTGGTT TGGTA	GTGCACATGGTGGAGT CAGGA
*IFIH1*	AACAGCAACATGGGC AGTGA	TGGCTGGGCAACTTC CATTT

### 2.6 Construction of TF-mRNA-miRNA network

Transcription factors (TFs) play a critical role in the regulation of gene expression (30). Transcription Factor Enrichment Analysis (TFEA) (31) prioritizes TFs based on the overlap between a provided list of genes and previously annotated TF targets from various sources. ChEA3 (https://maayanlab.cloud/chea3/) (32) is a web-based TFEA tool. By entering the overlapping genes of two diseases into the ChEA3 website and selecting the top 10 TFs by Mean Rank value, we can identify the important TFs for the overlapping genes in the two diseases. The Enrichr (https://maayanlab.cloud/Enrichr/) (33) website is a tool for gene set enrichment analysis based on multiple databases. Among its resources, the Enrichr Submissions TF Gene Co-occurrence database integrates association data between TFs and target genes from various studies, supporting the analysis of co-occurrence relationships between TFs and genes (33). MicroRNA (miRNA) is a type of endogenous non-coding small RNA that can cause degradation of target mRNA or inhibit its translation (34). MiRWalk (http://mirwalk.umm.uni-heidelberg.de/) (35) predicts miRNA-target interactions by integrating known data and literature, combined with a random forest algorithm. MiRTarBase (https://awi.cuhk.edu.cn/~miRTarBase/miRTarBase_2025/php/index.php) (36) is a large biological database that primarily provides experimentally validated miRNA-target interactions. TarBase (https://dianalab.e-ce.uth.gr/tarbasev9) (37) is a reference database specifically dedicated to collecting and managing experimentally validated miRNA target data. Using the miRWalk database, we predict potential miRNA targets within the 3’ untranslated region (3’UTR), 5’ untranslated region (5’UTR), and coding sequence (CDS) of the overlapping genes. The key targets were imported into the miRTarBase and TarBase databases to identify experimentally validated miRNAs that interact with the key targets. The intersecting miRNAs obtained from the three databases were considered to significantly affect the key targets and possess high credibility. Finally, we construct a network diagram illustrating the interactions between TFs, miRNAs and key targets. The network of TF-mRNA-miRNA interactions is visualized using Cytoscape version 3.10.0.

### 2.7 Drug prediction and molecular docking

The Drug Signatures Database (DSigDB) (38) is a database that allows drug enrichment analysis based on lists of genes, with the goal of screening for potential therapeutic drugs (38). Using the Enrichr platform based on DSigDB, we entered 58 overlapping genes between SLE and ME/CFS and ranked them according to their adj. pValue. From this analysis, we extracted the top 10 potential drug for the treatment of SLE-associated fatigue.

Molecular docking studies were conducted to assess the binding affinities between the top five potential drugs and key targets. The three-dimensional conformations of the drugs were obtained from the PubChem (https://pubchem.ncbi.nlm.nih.gov/) compound repository, while protein structures corresponding to the key targets were obtained from the Protein Data Bank (https://www.rcsb.org/). Binding affinities were evaluated using the AutoDock computational tool. Subsequently, the resulting drug-target complexes were visualized for further examination.

## 3 Results

### 3.1 Collecting overlapping genes between SLE and ME/CFS

**Figure 2:**
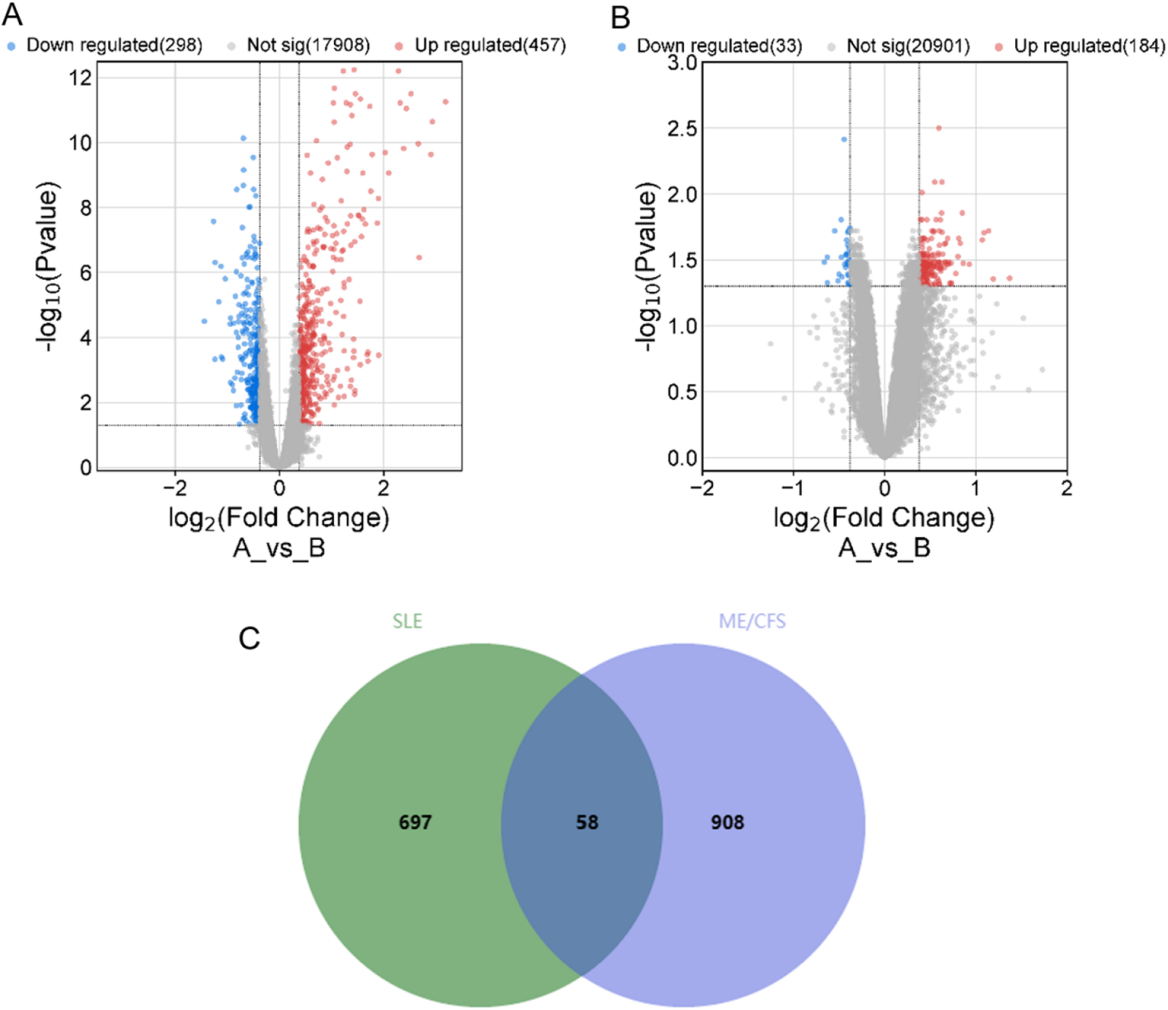
Volcano plot for 2 datasets and Venn diagram of overlapping genes between SLE and ME/CFS. **(A)** Volcano plot of GSE72326. **(B)** Volcano plot of GSE14577. **(C)** Venn diagram of the overlap DEGs between SLE and ME/CFS. The identified 58 common genes that may be related to their shared pathogenic mechanisms.

By searching the GEO, DisGeNET, and GeneCards databases, we collected genes associated with SLE and ME/CFS. In GSE72326, we obtained 755 DEGs associated with SLE (**Figure 2A**). However, the 217 DEGs in GSE14577 for ME/CFS are relatively few (**Figure 2B**). To improve the credibility of the data, we obtained 118 and 817 ME/CFS-related genes from DisGeNET and GeneCards databases, respectively. After removing duplicates in the three databases, we had 966 genes associated with ME/CFS. Finally, by overlapping related genes of SLE and ME/CFS, we identified 58 common genes as overlapping genes and visualized them using a Venn diagram (**Figure 2C**).

### 3.2 Analysis of GO and KEGG

**Figure 3:**
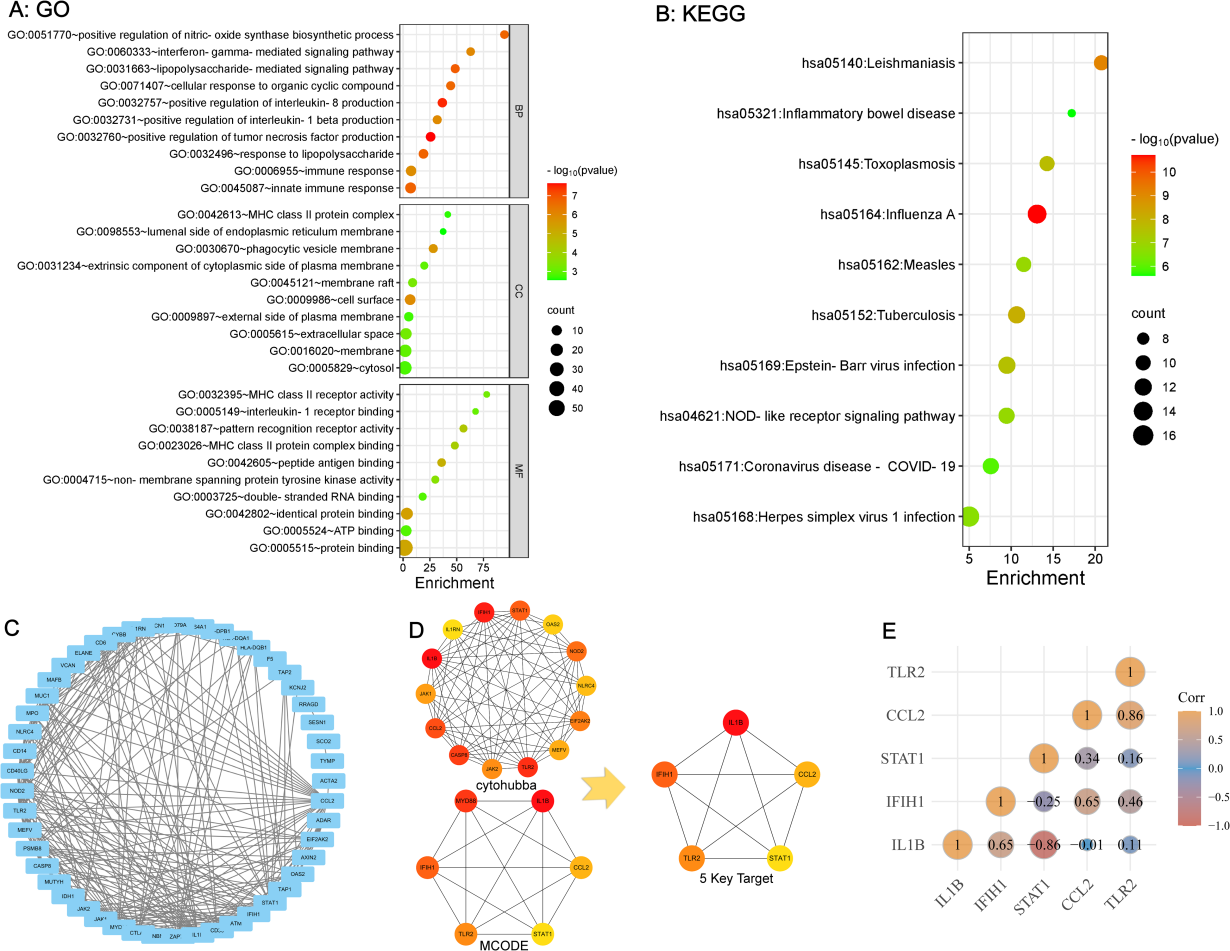
GO and KEGG enrichment analysis of overlapping genes and identification of key targets **(A, B)** Strong association with innate immune response and inflammation based on GO and KEGG Pathway Enrichment. **(C)** PPI Network Analysis of overlapping Genes Between SLE and ME/CFS. **(D)** Identification of 5 key targets. **(E)** Correlation Analysis Among 5 key Targets.

The DAVID website was utilized to analyze the 58 overlapping genes. The enrichment data for GO and KEGG were ranked according to FDR-values, with the top 10 selected as significant entries. **Figure 3A** illustrates the outcomes of GO analysis, encompassing the three terms: BP, CC, and MF. **Figure 3B** shows the findings derived from the KEGG analysis. Pathways related to innate immune response and inflammation were significantly enriched, including those associated with LPS, TNF, IL-8, IFN-γ, IL-1β, and MHC-II.

### 3.3 Obtaining key targets and performing correlation analysis

The STRING platform was used for PPI network analysis of the 58 overlapping genes, resulting in a protein interaction graph with 49 nodes and 243 edges. The results were then visualized using Cytoscape version 3.10.0 (**Figure 3C**). Within Cytoscape, the MCODE plugin was implemented to analyze the protein network and construct a key targets module containing 14 common genes. In addition, the cytoHubba plugin in Cytoscape was used to analyze the protein network, from which the top six targets with the highest scores were extracted. By overlapping the targets identified by both plugins, five key targets for SLE and ME/CFS were determined (**Figure 3D**): *IL1β, CCL2, TLR2, STAT1, IFIH1*. The correlation analysis between these five key targets is shown in **Figure 3E**. The ROC analysis results for the five key targets across the two diseases are summarized in **Table 2**. The ROC curves for each key target are shown in **Supplementary Data Sheet 1**.

**Table 2:** ROC analysis of key targets in SLE and ME/CFS.

Key Target	AUC of ROC (95% CI)	
	SLE	ME/CFS
**IL1β**	0.64 (0.43, 0.85)	0.75 (0.55, 0.95)
**CCL2**	0.76 (0.58, 0,93)	0.67 (0.45, 0.89)
**TLR2**	0.89 (0.75, 1)	0.75 (0.55, 0.95)
**STAT1**	0.90 (0.78, 1)	0.79 (0.59, 0.98)
**IFIH1**	0.96 (0.88, 1)	0.76 (0.55, 0.97)

This table presents the area under the ROC curve for five key targets in SLE and ME/CFS. The 95% confidence interval (CI) of the area under the curve (AUC) is provided in brackets.

### 3.4 Expression of key targets in single-cell clusters

**Figure 4:**
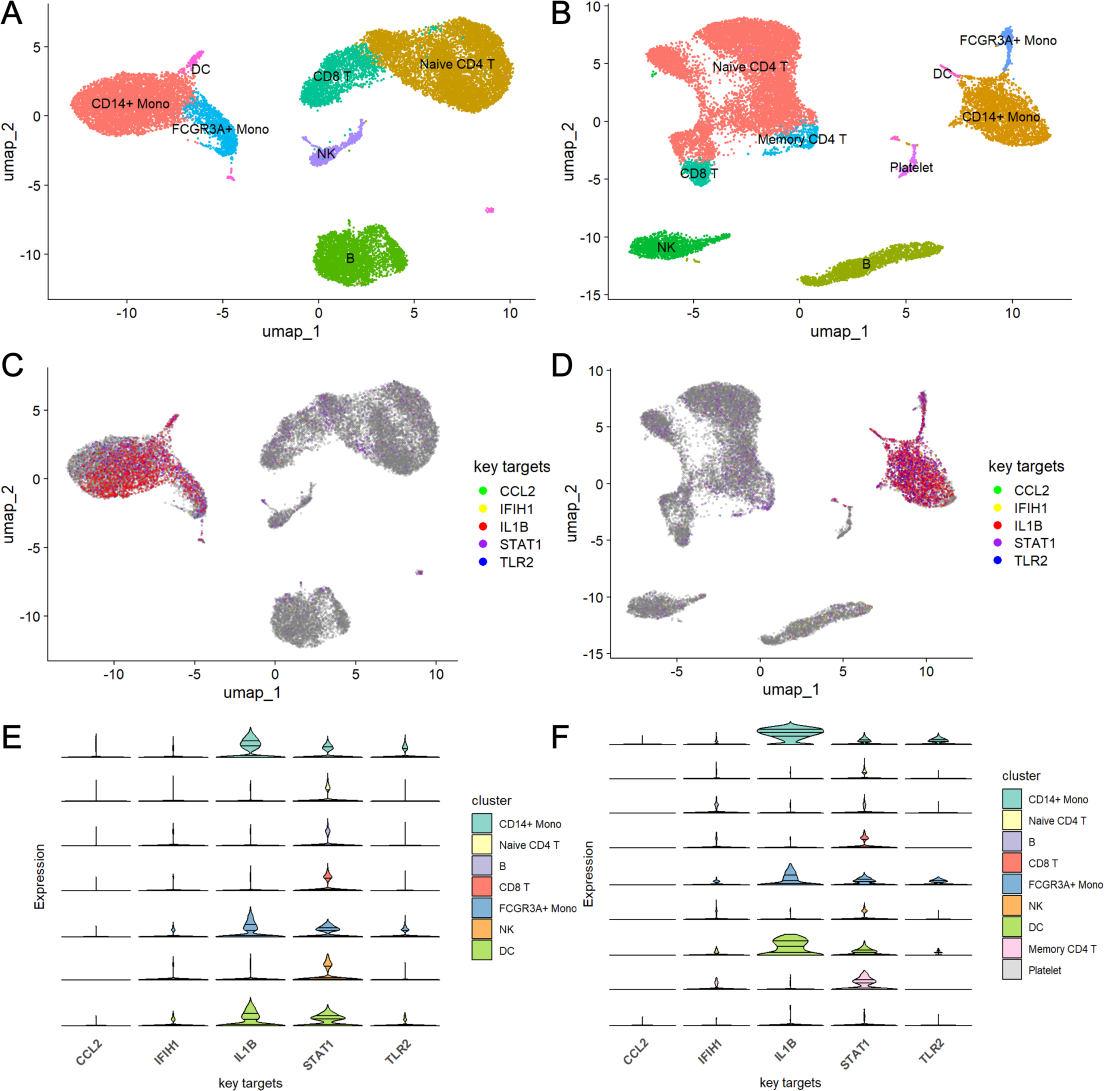
UMAP and violin plot analysis of key target gene expression across cell types in SLE and ME/CFS samples **(A, B)** UMAP plots of single-cell RNA sequencing data from SLE **(A)** and ME/CFS **(B)** samples. **(C, D)** Expression distribution of five key target genes across cell clusters in SLE **(C)** and ME/CFS **(D)** samples. **(E, F)** Violin plots of the expression levels of five key target genes across cell types in SLE **(E)** and ME/CFS **(F)** samples.

**Figure 5:**
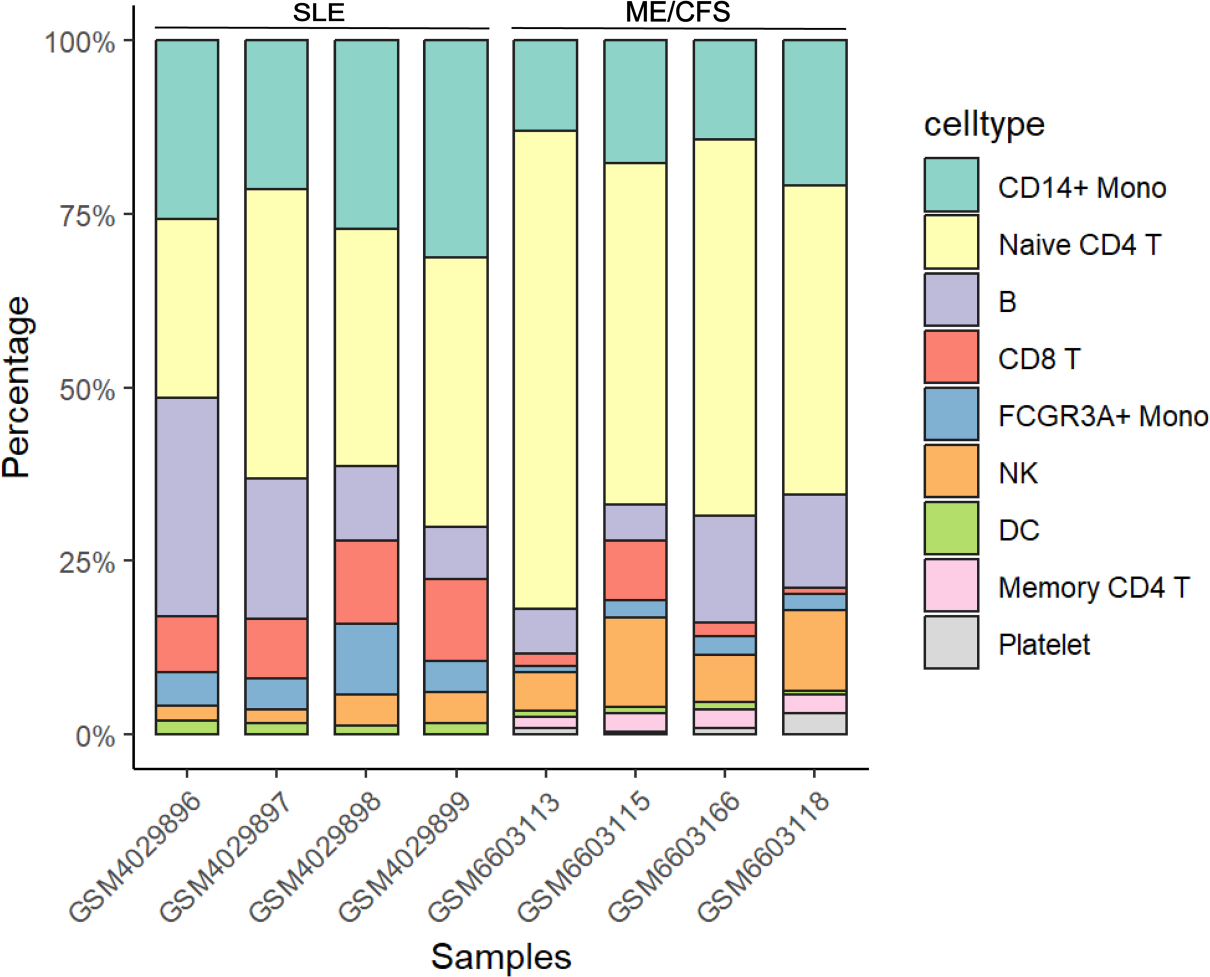
Composition of cell type in SLE and ME/CFS samples.

Single-cell RNA sequencing analysis of datasets GSE135779 for SLE and GSE214284 for ME/CFS identified distinct immune cell populations across both disease conditions. UMAP visualization (**Figures 4A, B**) revealed clusters of immune cell types, including CD14+ monocytes, FCGR3A+ monocytes, dendritic cells (DC), naive and memory CD4+ T cells, CD8+ T cells, B cells, NK cells, and platelets. Each cell type showed a unique spatial distribution, with some differences in abundance across cell populations between SLE and ME/CFS. **Figures 4C, D** show the expression patterns of the five key targets across different cell types in SLE and ME/CFS samples, respectively. The UMAP visualizations reveal that the expression of these key targets is primarily concentrated within monocytes in both diseases. Violin plots (**Figures 4E, F**) showed the key target genes (*CCL2, IFIH1, IL1B, STAT1*, and *TLR2*) had differential expression patterns across these cell types. Notably, *IL1B* and *STAT1* showed elevated expression in CD14+ monocytes. Further analysis of cell-type composition (**Figure 5**) showed distinct differences in the percentage distributions of each cell type across samples for SLE and ME/CFS. In SLE samples, the proportion of CD8+ T and B cells is higher, while in ME/CFS, the main component is naive CD4+ T cells. At the same time, memory CD4+ T cells and NK cells are more prominent in ME/CFS samples. In contrast, SLE samples show a greater proportion of CD14+ monocytes and FCGR3A+ monocytes.

### 3.5 Key target expression under monocyte activation

**Figure 6:**
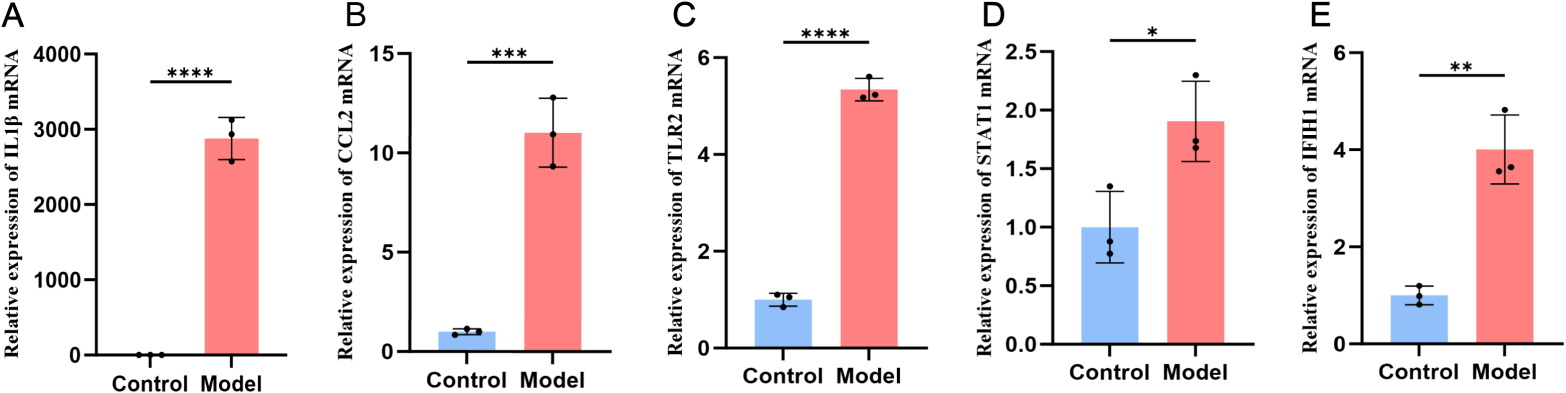
Expression of the five key targets in the THP-1 monocyte model group and the control group. The expression is elevated in *IL1β* **(A)**, *CCL2* **(B)**, *TLR2* **(C)**, *STAT1* **(D)**, *IFIH1* **(E)**.

Our experiments further validated the changes in key target expression in activated monocytes. We assessed mRNA expression levels using RT-qPCR. As shown in **Figure 6**, compared to the control group, mRNA levels of the five key targets—*IL-1β, CCL2, TLR2, STAT1*, and *IFIH1*—were significantly increased in cells treated with LPS for 24 hours, with IL-1β showing the most pronounced change.

### 3.6 Construction of TF-mRNA-miRNA network

**Figure 7:**
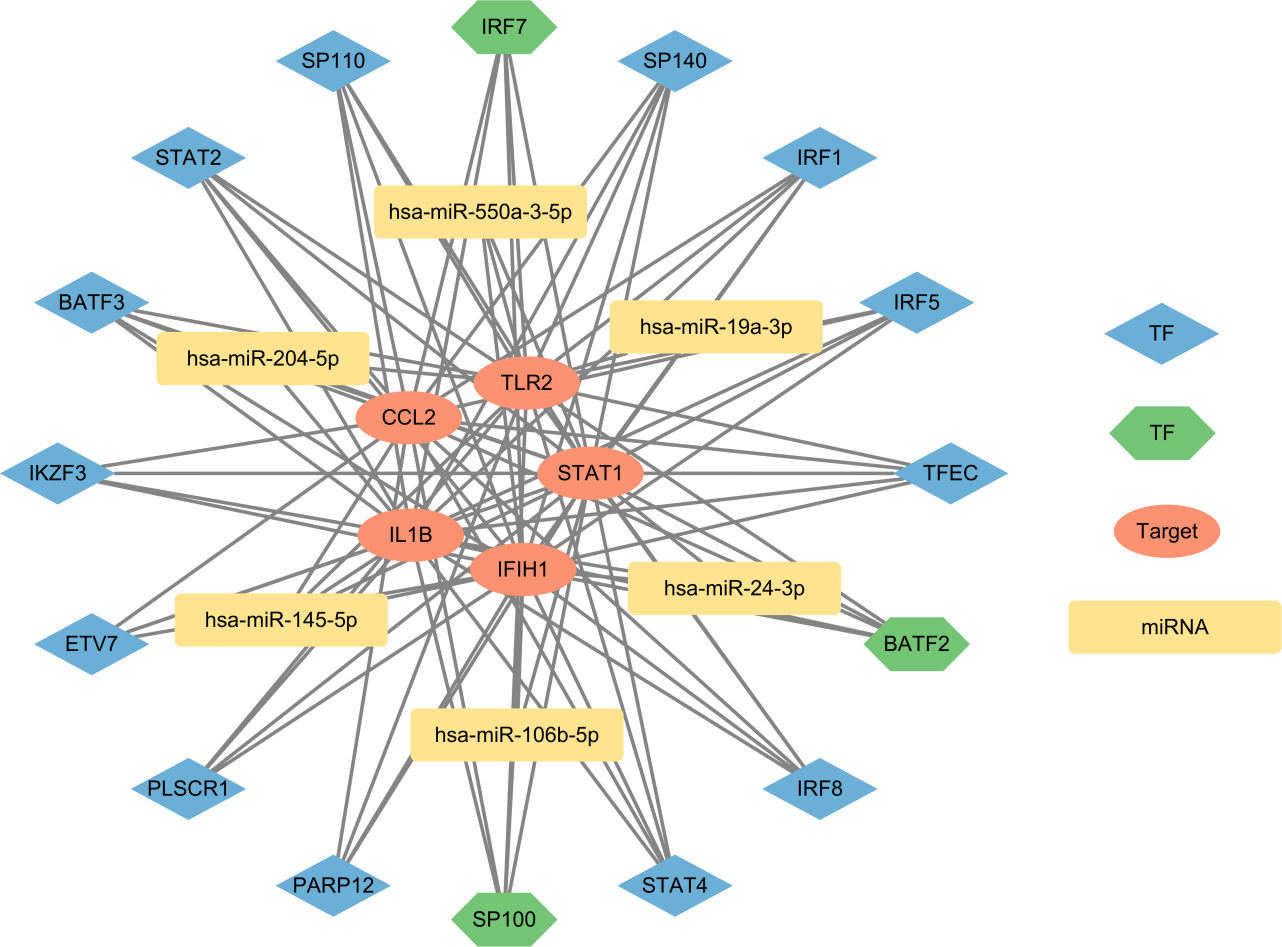
The network presents the TF-mRNA-miRNA coregulatory network. The network consists of 27 nodes and 93 edges including 16 TF-genes, 6 miRNA and 5 key targets. The nodes in red color are the key targets, blue and green nodes represent TF-genes and yellow nodes indicate miRNA. The green TFs (BATF2, IRF7, SP100) represent results obtained from both databases, indicating a higher level of credibility.

After entering the 58 overlapping genes of the two diseases into the ChEA3 and Enrichr website, 10 TFs were obtained from each database. Among them, the Enrichr website identified one TF, STAT1, which overlaps with the key targets, so it was excluded. Taking the union of the two sets of TFs, a total of 16 TFs were obtained, with three being duplicate TFs: BATF2 (basic leucine zipper ATF-like transcription factor 2), IRF7 (interferon regulatory factor 7), and SP100 (SP100 nuclear antigen). The five key targets were imported into the miRTarBase, TarBase, and miRWalk databases, resulting in 54, 189, and 891 miRNAs, respectively. After taking the intersection, a final set of six miRNAs was obtained: hsa-miR-204-5p, hsa-miR-24-3p, hsa-miR-106b-5p, hsa-miR-19a-3p, hsa-miR-145-5p, and hsa-miR-550a-3p. These miRNAs are considered to significantly affect the key targets. The interactions between TFs and miRNAs, derived from the overlapping genes, were represented alongside the key targets using Cytoscape version 3.10.0 to establish the regulatory network diagram. This co-regulatory network consists of 27 nodes and 93 edges, with 16 TFs and 6 miRNAs interacting with the 5 key targets (**Figure 7**).

### 3.7 Drug prediction and molecular docking

Using the DSigDB database through the Enrichr platform, drug predictions were performed for the 58 overlapping genes. The top 10 potential drugs were selected based on the adj. pValue and the results are presented in **Table 3**.

**Table 3:** Medicine predictions based on the DSigDB database.

Medicines	Mechanism	Genes involved	adj. *p*Value
Suloctidil	Vascular relaxing activity; calcium antagonist; antithrombotic agent	*IL1RN,GCH1,STAT1,TAP2,EIF2AK2,TAP1,ADAR,TYMP,IFIH1,MAFB,OAS2,CCL2,MYD88*	2.55E-13
N-Acetyl-L-cysteine	Antioxidant; precursor to glutathione, reduces oxidative stress and modulates immune response.	*ABCB1,STAT1,MPO,TYMP,ACTA2,CASP8,IL1B,CCL2,ATM,NBN,CD14,TLR2,JAK1*	4.23E-09
Simvastatin	Statin; inhibits cholesterol synthesis and has anti-inflammatory effects.	*ACTA2,ABCB1,CASP8,CD40LG,STAT1,IL1B,IDH1,CTLA4,CCL2,ATM,MPO,TLR2*	3.52E-08
ACMC-20mvek	Predicted compound; mechanism unclear.	*ABCB1,CASP8,CD40LG,IL1B,CCL2,CD14,TLR2,F5*	2.23E-07
Camptothecin	Topoisomerase I inhibitor; induces DNA damage, mainly anticancer.	*ABCB1,CASP8,IL1B,TAP1,CCL2,ATM,NOD2,JAK2*	6.29E-07
Nitric oxide	Signaling molecule; modulates vasodilation and immune response.	*CASP8,GCH1,STAT1,IL1B,CYBB,JAK2,MPO,MYD88,TLR2*	0.000001239
Cardidigin	Digitoxin, cardiac glycoside, increasing calcium, inhibits Na^+^/K^+^-ATPase	*ABCB1,CASP8,IDH1,IL1B,CCL2,NOD2*	0.000001285
Fenbendazole	Anthelmintic; disrupts microtubules, with emerging immune effects.	*ABCB1,STAT1,IDH1,CCL2,JAK2*	0.000001662
ZINC SULFIDE	Mechanism unclear. Zinc ions are involved in the regulation of innate and adaptive immune responses.	*IL1RN,IL1B,CCL2,NLRC4,CD14,MYD88*	0.000001662
GNF-Pf-78	Predicted compound; mechanism unclear.	*STAT1,IDH1,IL1B,NOD2,JAK2*	0.000004717

**Figure 8:**
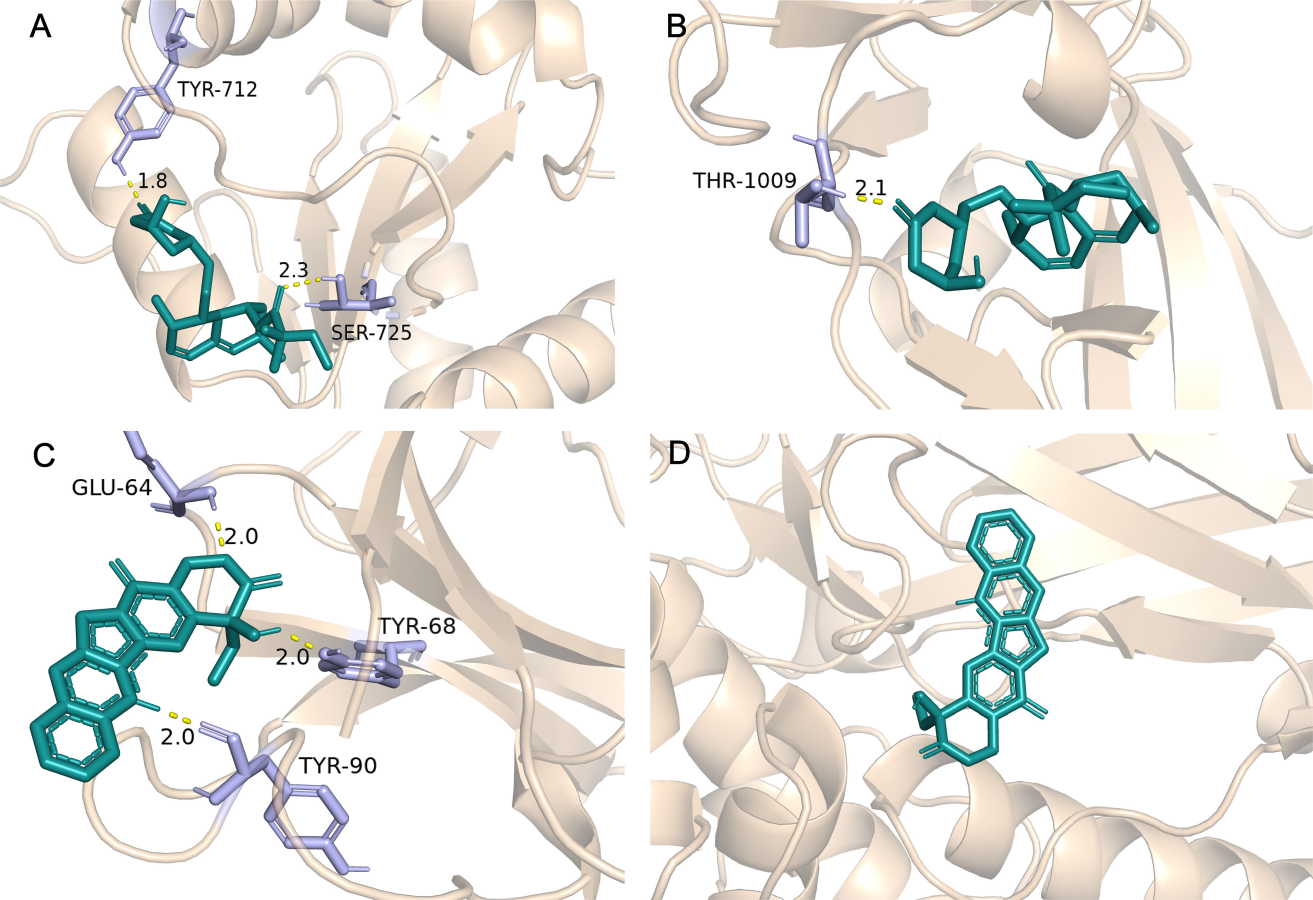
Partial molecular docking results. **(A)** Depicts the docking interaction between Simvastatin and TLR2. **(B)** Depicts the docking interaction between Simvastatin and IFIH1. **(C)** Depicts the docking interaction between camptothecin and IL1B. **(D)** Depicts the docking interaction between camptothecin and STAT1.

Molecular docking was performed on the top five predicted drugs with 5 key targets using AutoDock computational tool. The binding energy reflects the interaction potential between the drug and the target; the lower the binding energy, the greater the affinity and stability of the drug-target complex. Among them, N-Acetyl-L-cysteine (NAC) and camptothecin show a strong binding effect on IL1B. Additionally, simvastatin and camptothecin also have good binding effects on CCL2, TLR2, STAT1, and IFIH1. More binding energies (in kcal/mol) are showed in **Table 4**. The more favorable molecular docking results were visualized using PyMOL (in **Supplementary Data Sheet 2**), with some of the results depicted in **Figure 8**.

**Table 4:** Therapeutic efficacies and molecular affinities of medicines.

Medicine	Efficacy	Molecular affinity (kcal/mol)				
		IL1B	CCL2	TLR2	STAT1	IFIH1
Suloctidil	Vasodilation	-4.21	-4.79	-5.51	-2.51	-5.5
N-Acetyl-L-cysteine	Management of acetaminophen overdose, mucolytic activity	-5.09	-3.45	-3.36	-2.91	-3.44
Simvastatin	Lipid lowering, atherosclerosis prevention	-4.8	-6.64	-6.94	-4.11	-8.72
ACMC-20mvek	Unreported	-4.8	-6	-5.63	-2.94	-7.5
Camptothecin	Antineoplastic therapy	-6.04	-6.24	-6.6	-5.37	-8.55

## 4 Discussion

SLE and ME/CFS are two distinct diseases, but they share certain commonalities. Patients of both frequently experience fatigue, for which there are limited options available (39). Immune dysregulation may serve as a bridge connecting the two, although the specific extent and categories remain unclear. This study aims to elucidate the shared mechanisms between the two conditions and provide potential options for clinical management.

The 58 overlapping genes identified showed a strong correlation with innate immunity and inflammation in GO term and KEGG. Specifically, the BP highlights the role of innate immunity and inflammatory factors (such as TNF, IL-8, IFN-γ, IL-1β, etc.) in SLE and ME/CFS. Research supports the importance of innate immunity and inflammatory factors in SLE. For example, abnormalities in the phagocytic function of macrophages can lead to the exposure of autoantigens and the initiation of autoimmune responses (40). Inflammatory factors like TNF-α and IL-8 are elevated in SLE patients, leading to apoptosis in various cells, resulting in the release of nucleo-cytoplasmic contents (41–43). In the context of ME/CFS, components of the innate immune system, such as natural killer cells, DCs, and monocyte as well as pro-inflammatory cytokines (TNF-α and IFN-γ), also exhibit aberrant behavior (44). Specifically, NK cells exhibit reduced cytotoxic activity (45), and the number of plasmacytoid DCs is also significantly decreased, which may lead to lower levels of type I interferon and hinder the effective clearance of pathogens in ME/CFS patients (44). Classic monocytic dysregulation has also been validated in ME/CFS patients, suggesting that inappropriate differentiation and tissue migration may also be contributing factors (19). Dysregulation of the cytokine network further supports the immune dysfunction in ME/CFS (46), with elevated levels of TNF-α and IL-1β observed in ME/CFS patients (47, 48). Another study reported increased TNF-α in fasting plasma from 53 patients, which correlated with the severity of fatigue (49). The abnormal levels of these factors may induce fatigue through various mechanisms, including the regulation of indoleamine 2,3-dioxygenase by inflammatory factors, potentially explaining the observed fatigue symptoms in SLE and ME/CFS (50–53). The analysis of CC, MF, and KEGG suggests an etiological link between SLE and ME/CFS, which includes viral and bacterial infections, as well as variations in subsequent downstream pathways.

To further identify key targets, based on the PPI network, *IL1β, CCL2, TLR2, STAT1*, and *IFIH1* were declared as key targets due to their high degrees of connectivity. IL-1β, secreted by monocytes, macrophages, and DCs, exerts functions in peripheral immunity. Additionally, it can also impact the dopaminergic and serotonergic neurotransmission systems in brain (54). During the acute phase of ME/CFS, the concentration of IL-1β is significantly correlated with fatigue symptoms (54). CCL2, also known as monocyte chemoattractant protein (MCP-1), acts as an attractant for monocytes and serves as an inflammatory mediator. Its concentration is correlated with symptoms such as fatigue (55), and the expression of CCL2 is elevated in blood samples from ME/CFS patients (56). TLR2 is a membrane protein that can recognize pathogens and induce cytokine production. Its activation may be related to the pathophysiological mechanisms of immune-related chronic fatigue triggered by various diseases (57, 58). The protein MDA5, encoded by the *IFIH1* gene, is involved in the identification of virus (59). It has been reported that 64% of patients consider infection as a peri-event (60), and our findings once again emphasize the particular significance of the anti-pathogen pathway. STAT1 is a key signaling molecule activated by IFN-α/β and IFN-γ (61). Its expression is upregulated in SLE (62). Increased expression of the *IFIH1* gene in SLE patients promotes the production of type I IFN and various cytokines, which significantly impact the onset and severity of SLE (63, 64). Additionally, correlation analysis among the key targets in SLE reveals a significant negative correlation between STAT1 and IL-1β. The mechanism may be related to IL-1β inhibiting IFNα/β-induced STAT1 phosphorylation through the proteasome (65, 66). The antagonism between these two cytokines characterizes the heterogeneity of inflammatory responses. Alterations in their dominant roles may lead to different inflammatory properties and damage (66). In SLE, ROC analysis for *STAT1* and *IFIH1* demonstrated superior performance, while *TLR2* and *CCL2* showed moderate discriminatory ability. In ME/CFS, ROC analysis for *IL1β*, *TLR2*, *STAT1*, and *IFIH1* had moderate discriminatory ability. This indicates that these targets can distinguish the occurrence of the diseases to some extent, providing valuable insights for identifying specific biomarkers of fatigue associated with both diseases. In conclusion, key targets reveal the specifical inflammation mechanism underlying the fatigue in SLE and ME/CFS.

To validate the expression of key targets at the single-cell level, we utilized public datasets for analysis. Dimensionality reduction and clustering identified specific populations in the peripheral blood samples of SLE and ME/CFS. The higher proportion of CD8+ T and B cells in SLE samples reflects its unique disease context, while the identification of memory CD4+ T cells in ME/CFS may be related to the patients’ history of infections. The expression patterns of key targets strongly suggest that the monocytic population may be involved in the shared pathological processes of both conditions. Classic monocytic dysregulation (CD14+) has been revealed in ME/CFS patients. GSEA of the proteomics data confirmed an increased activation state of classic monocytes in ME/CFS, with scores from the diseased cells correlating with multidimensional fatigue scale scores of patients (19). Although non-classic monocytes (CD16+, i.e., FCGR3A+) have been shown to be associated with inflammation in SLE (67), classical monocytes, which are major producers of TNF-α and IL-1 in response to LPS, still play a significant role in inflammatory effects, especially under harmful stimuli (68). The results indicate that the proportion of classic monocytes in SLE is higher than in ME/CFS, suggesting that SLE patients also experience profound monocytic dysregulation, which may bridge the connection between immune dysregulation and fatigue.

THP-1 is a human leukemia-derived monocyte cell line that is CD14-positive and CD16-negative (69). Under the influence of PMA, THP-1 cells exhibit adhesion and differentiation processes similar to those of classical monocytes (70). In the biological processes identified by GO analysis, LPS pathway ranks third in enrichment, representing a shared mechanism between SLE and ME/CFS. Therefore, we selected LPS as an inflammatory stimulus for further experiments. While single-cell analysis did not show notable expression of CCL2 and IFIH1 in CD14+ monocytes, RT-qPCR results indicated that all five key targets had significantly increased expression in LPS-incubated THP-1 cells compared to the control group. We believe this may relate to monocyte system distribution, as CCL2 creates a strong concentration gradient to attract monocytes (71). Activated monocytes/macrophages are more likely to migrate to tissues rather than remain in peripheral blood, potentially explaining the observed differences. This tendency was also noted in a study by Luyen et al., where classical monocytes from ME/CFS patients showed a propensity for tissue migration and macrophage differentiation (19). However, further research is needed to confirm classical monocyte dysregulation in SLE patients.

In addition, we explored the intrinsic regulatory mechanisms of the key targets. TFs are proteins that bind to specific DNA sequences and regulate the transcription of genetic information (72). MiRNAs, which are approximately 22-nucleotide-long non-coding small RNAs, participate in silencing the expression of target genes by degrading mRNA or inhibiting translation (73, 74). We selected the top 10 TFs from two databases to construct the network. Among them, BATF2, IRF7, and SP100 are considered high-confidence TFs. BATF2 has immune regulatory functions (75) and is associated with IRF1, playing a role in the inflammatory response in macrophages induced by IFN-γ and LPS activation (76). IRF7 is a interferon regulatory factor that respond to the activation of pattern recognition receptors (PRRs) by immune complexes in SLE (77). SP100 can limit the replication of many clinically significant DNA viruses and is an important component of the innate immune response (78). PLSCR1 and IKZF3 are associated with SLE thrombosis and disease risk (79, 80). In ME/CFS, these TFs have been found to be upregulated or downregulated to varying degrees, with their specific significance still to be explored (81–83). We predicted the miRNAs regulating the key targets using the miRWalk database and validated them in the MiRTarBase and TarBase experimental databases. The predicted miRNAs have been shown to play roles in cellular apoptosis, tumor suppression, and the development of SLE (84–89). The reduced cytotoxic activity of NK cells may be related to the differential expression of miRNAs, with miR-106, which is involved in cell proliferation, significantly decreased in NK cells (90). MiR-19a-3p has been found to inhibit M1 macrophage polarization by targeting *STAT1* and suppressing the STAT1/IRF1 pathway (91). MiR-204-5p has emerged as a key factor influencing M2 macrophage polarization (92). miR-145-5p may suppress cell proliferation in LPS-treated HUVECs by modulating macrophage polarization towards the M2 phenotype (93). The downregulation of m24 is considered essential during the differentiation of monocytes into macrophages, as it promotes the generation of functional macrophages and DCs (94). More relevant studies are needed to enrich the exploration in this field.

Finally, we predicted potential drugs and validated them through molecular docking. NAC is an antioxidant that promotes the synthesis of glutathione (95). A randomized controlled trial has shown that NAC can reduce the disease activity and complications of SLE (96). Another study indicated that SLE patients prescribed NAC (2.4g/day or 4.8g/day) experienced significant relief in both activity and fatigue symptoms (97), indirectly confirming our research findings. Other teams have also noted the potential role of NAC. A clinical trial (NCT04542161) on glutathione treatment for ME/CFS is currently underway and has entered the second phase, expected to conclude in 2025. Camptothecin is an antitumor agent that inhibits topoisomerase (98). Previous studies have demonstrated that low doses of camptothecin can significantly reduce lupus nephritis in mice (99) and exhibit anti-inflammation effect (100). The strong affinity for key targets suggests potential anti-inflammation effect, aligning with the strategy mentioned previously. However, camptothecin may pose significant toxicity risks, and efforts to mitigate its toxicity, along with further research, are needed to enhance the feasibility (101). Simvastatin has also shown promising results in molecular docking, its therapeutic effect on SLE and ME/CFS remains controversial (102). Lipid abnormalities are frequently observed in SLE, and statin therapy helps to reduce the risk of mortality and end-stage renal disease in these patients (103). Statins may contribute to fatigue symptoms by reducing the energy supply to muscle cells (102). Some case reports have also suggested that statins may have the unexpected effect of triggering SLE (104). Therefore, the use of statins should be cautious, as they may exert opposite effects. Furthermore, both suloctidil and ACMC-20mvek demonstrate affinity for the key targets. However, the clinical use of suloctidil has been constrained by reports of hepatotoxicity (105). As for ACMC-20mvek, being an experimental molecule, it requires appropriate molecular modifications and thorough toxicity validations before practical application.

In this study, we explored the shared molecular mechanisms between SLE and ME/CFS through bioinformatics analysis. Some limitations should be acknowledged. First, the small sample size of the ME/CFS dataset (GSE14577) with only 8 samples may reduce the robustness and statistical power of the differential expression analysis. This limitation may affect the generalizability and reliability of our findings. Second, our findings have not yet been validated in clinical samples, which may affect the robustness of the conclusions. Third, our analysis primarily relies on existing bioinformatics tools and databases. The predictive accuracy of these tools and the update frequency of the databases may impact the reproducibility of our study. The results of molecular docking alone cannot directly validate the effects of the predicted drugs, further *in vitro* and *in vivo* experiments are needed for verification. While these are shortcomings of our work, we believe they does not compromise the reliability of our conclusions.

## 5 Conclusion

In summary, we explored and identified overlapping genes in patients with SLE and ME/CFS, providing potential targets for fatigue in two diseases. Through a series of bioinformatics analyses, we obtained the key targets, BP, CC, MF and KEGG of overlapping genes, and TFs-mRNA-miRNA network. We also validated the expression of key targets in single-cell samples, and the results emphasize the potential involvement of monocytes in the fatigue symptoms of both diseases. Additionally, based on the overlapping genes, we predicted potential drugs for treating fatigue in SLE and ME/CFS. Combining molecular docking results with current clinical research, we propose that NAC and camptothecin are potential effective drugs. Our future work will deepen these findings, including an exploration of monocyte dysregulation related to fatigue in SLE and ME/CFS, as well as assessing drug efficacy in fatigue modulation. We believe that these findings have the potential to guide clinical trials and translational research in autoimmune conditions.

## Data Availability

Publicly available datasets were analyzed in this study. This data can be found here: http://www.ncbi.nlm.nih.gov/geo/; GSE72326; GSE14577, GSE211700, GSE128078, GSE135779, and GSE214284.

## Supplementary material

Supplementary Data Sheet 1 The ROC curves for each key target.

Supplementary Data Sheet 2 The molecular docking results.
