# Supplementary material for: Exploring the shared mechanism of fatigue between systemic lupus erythematosus and myalgic encephalomyelitis/chronic fatigue syndrome: monocytic dysregulation and drug repurposing
Source: Front Immunol. 2025 Jan 7;15:1440922. doi: 10.3389/fimmu.2024.1440922 (PMC11752880; doi:10.3389/fimmu.2024.1440922)

SLE-IL1 β

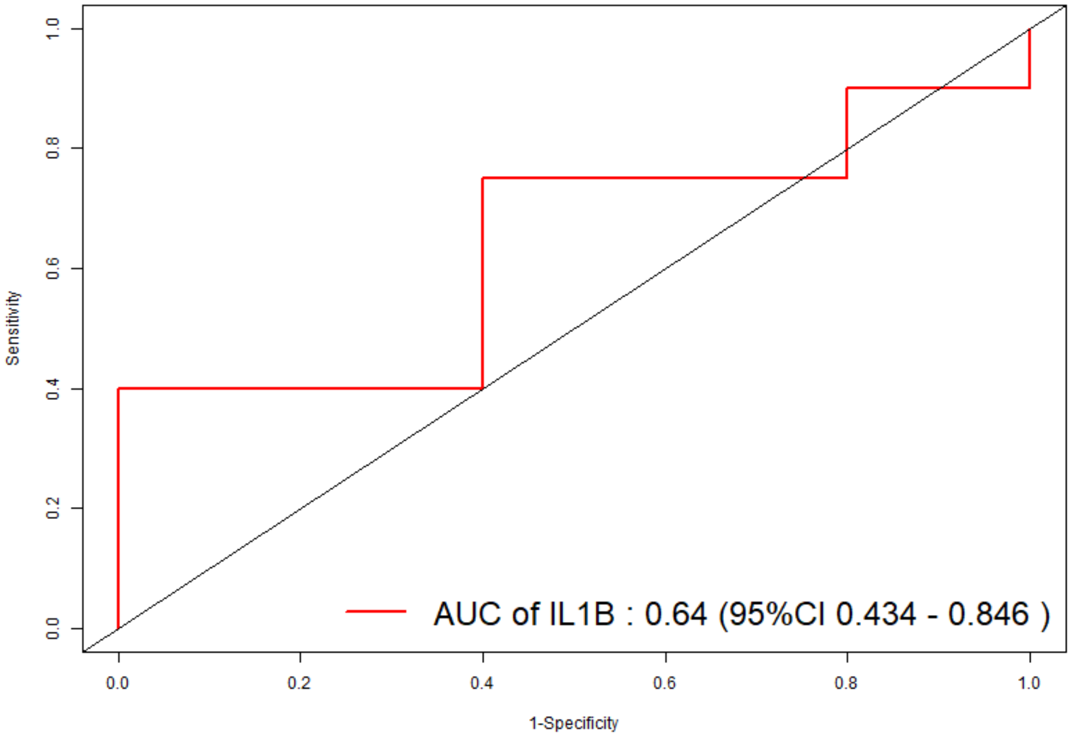

SLE-CCL2

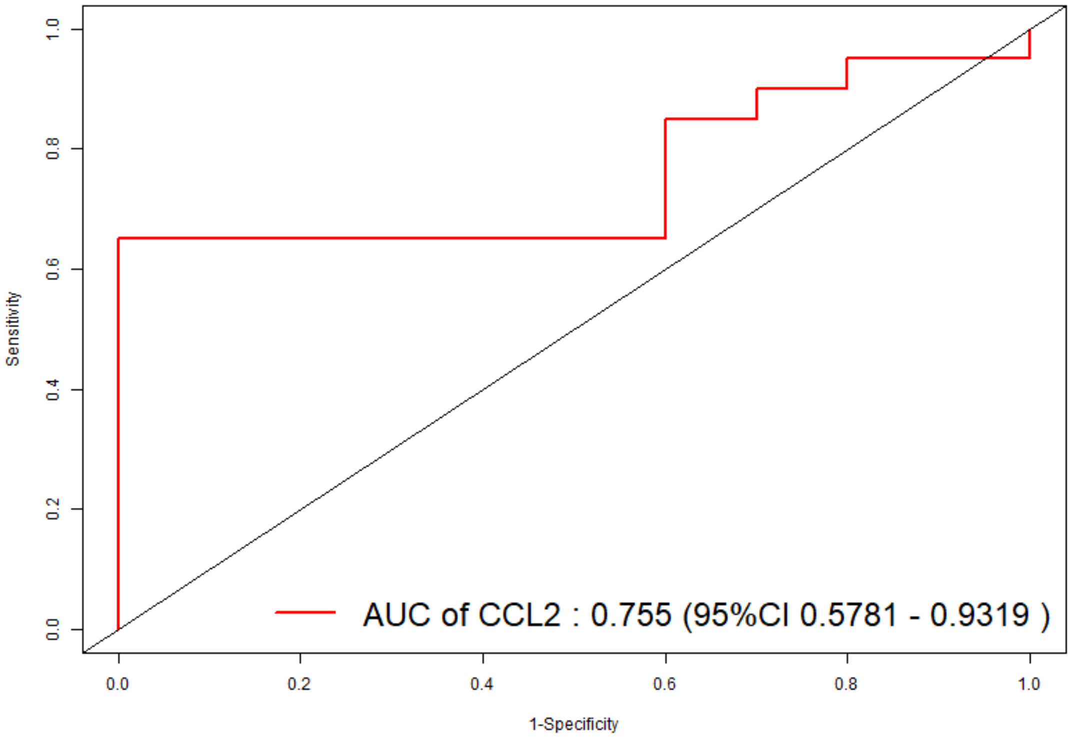

SLE-TLR2

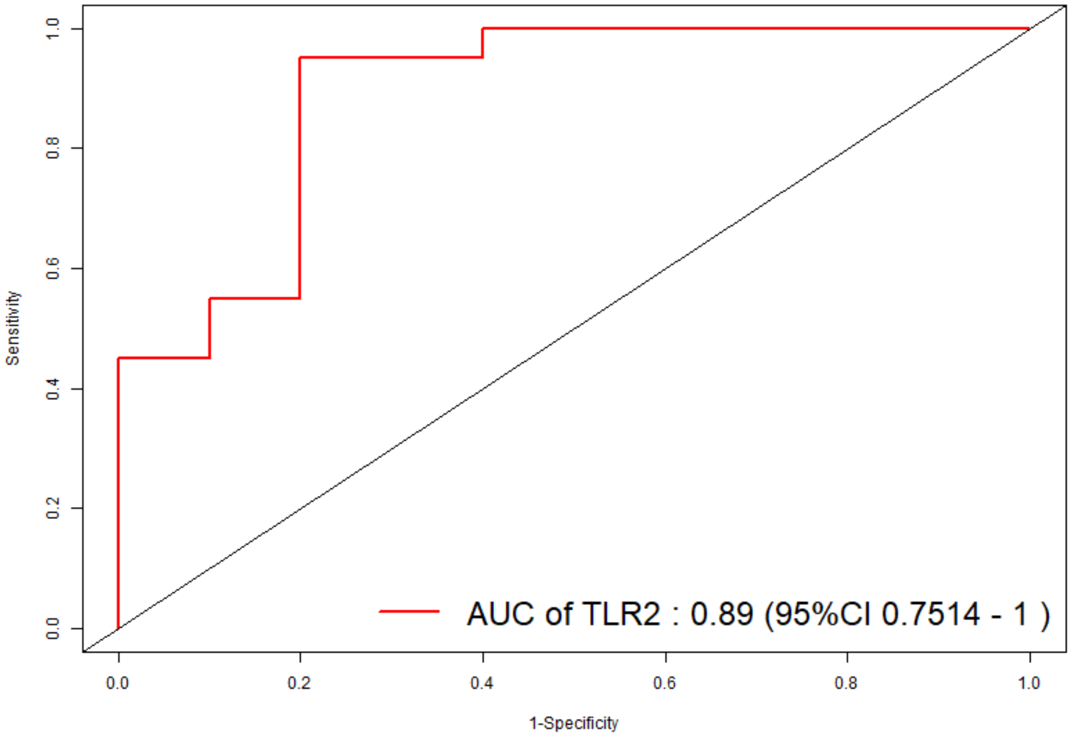

SLE-STAT1

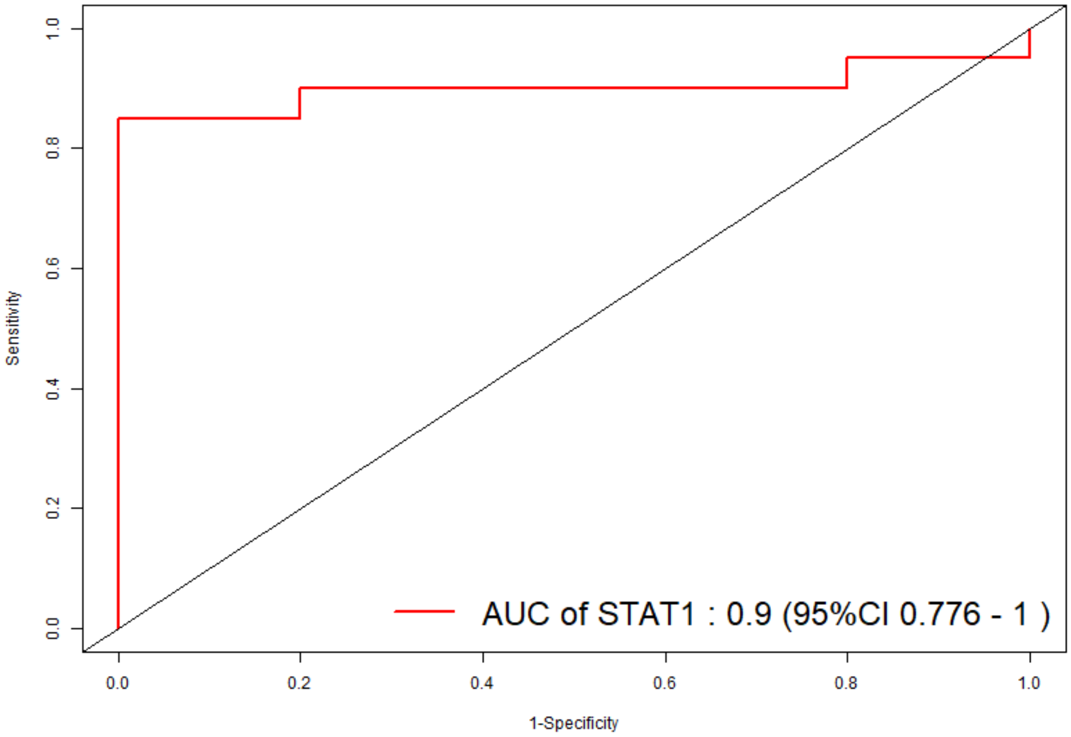

SLE-IFIH1

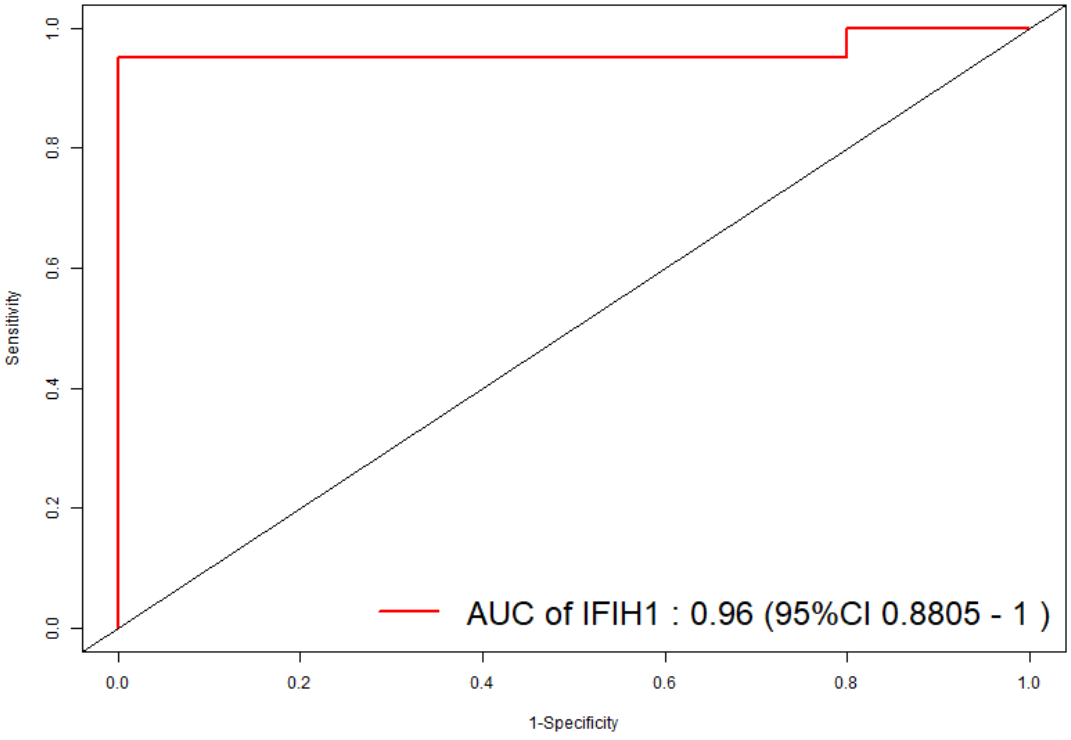

ME/CFS-IL1 β

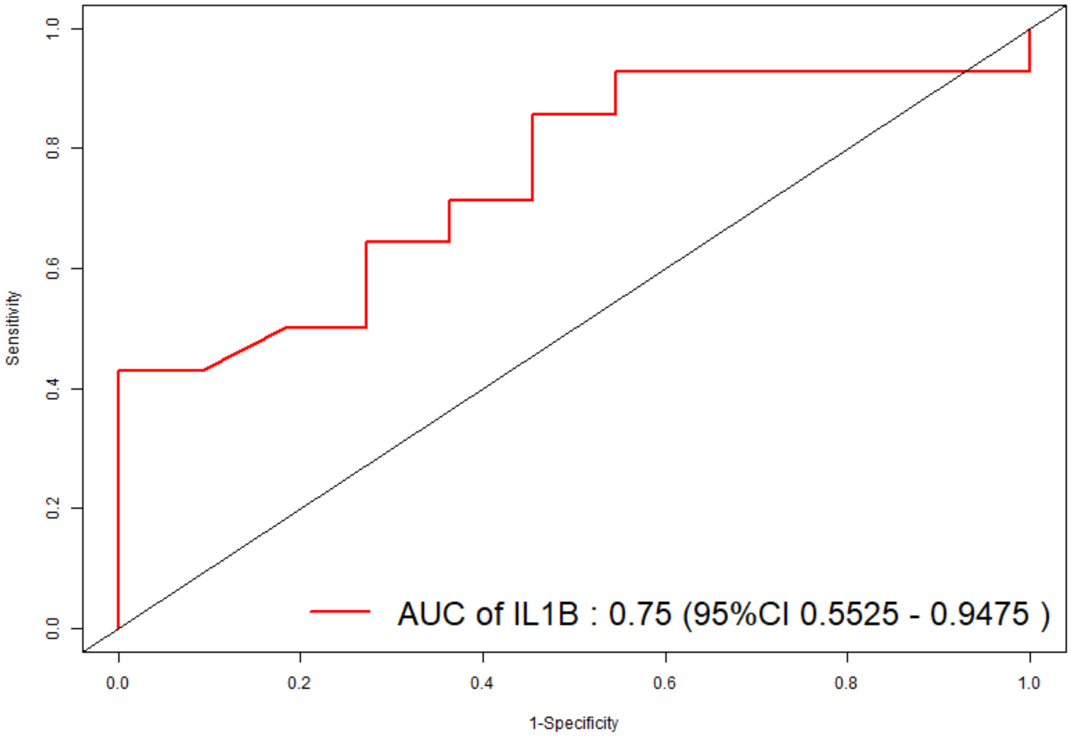

ME/CFS-CCL2

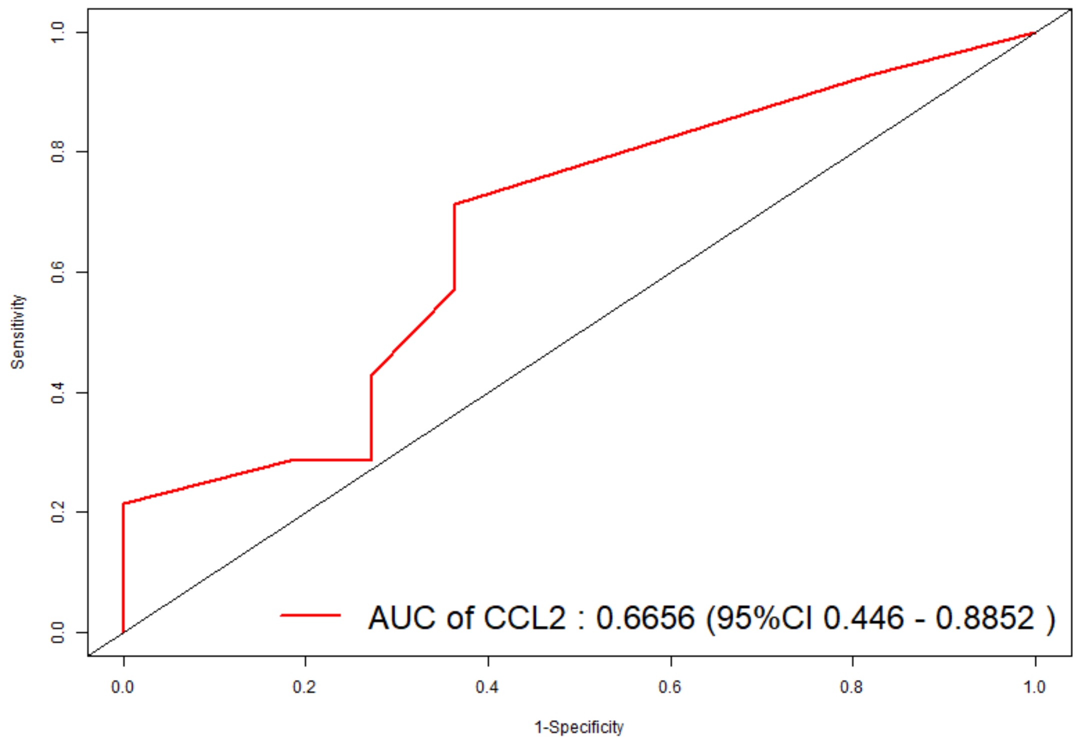

ME/CFS-TLR2

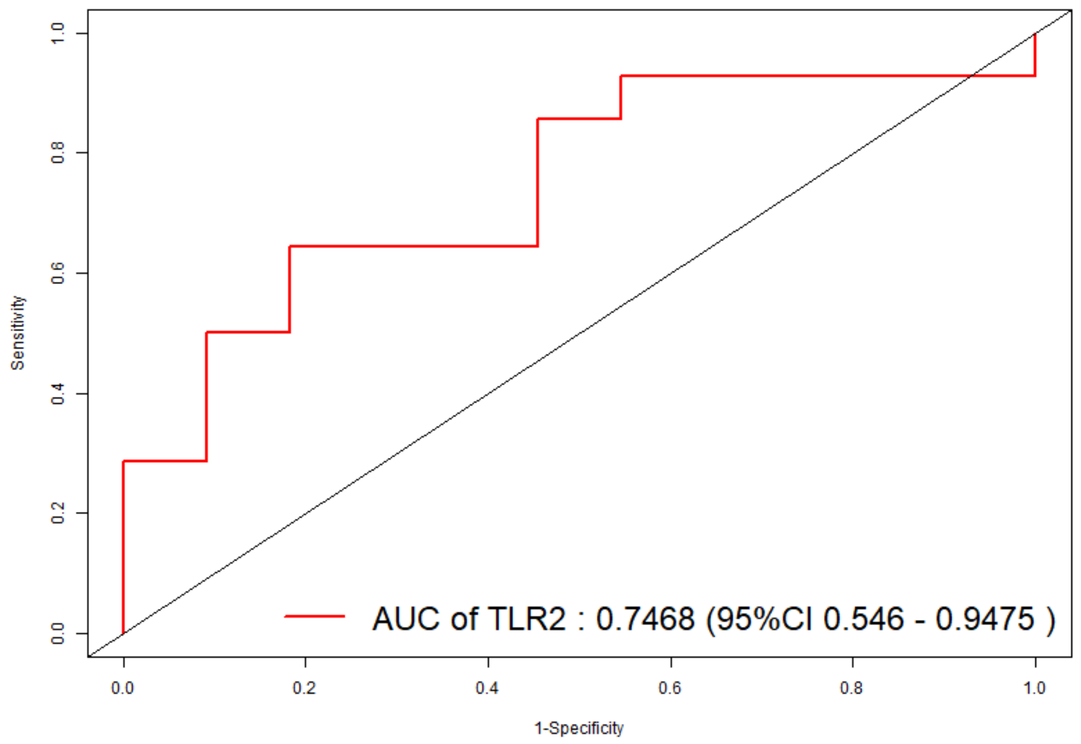

ME/CFS-STAT1

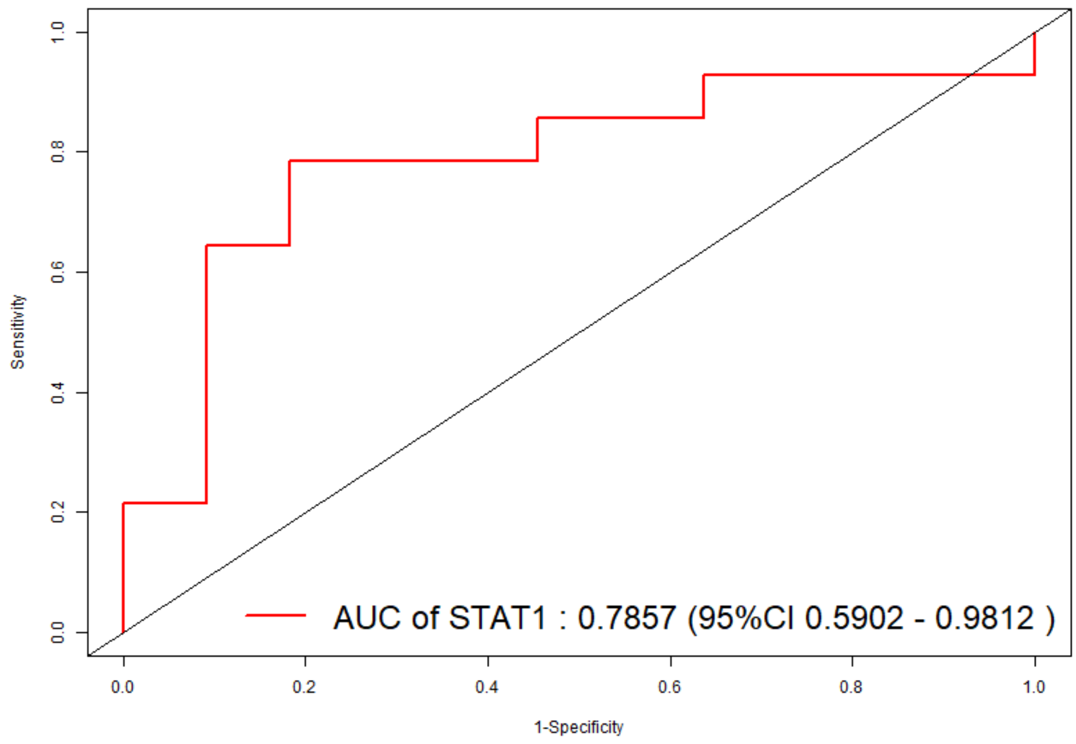

ME/CFS-IFIH1

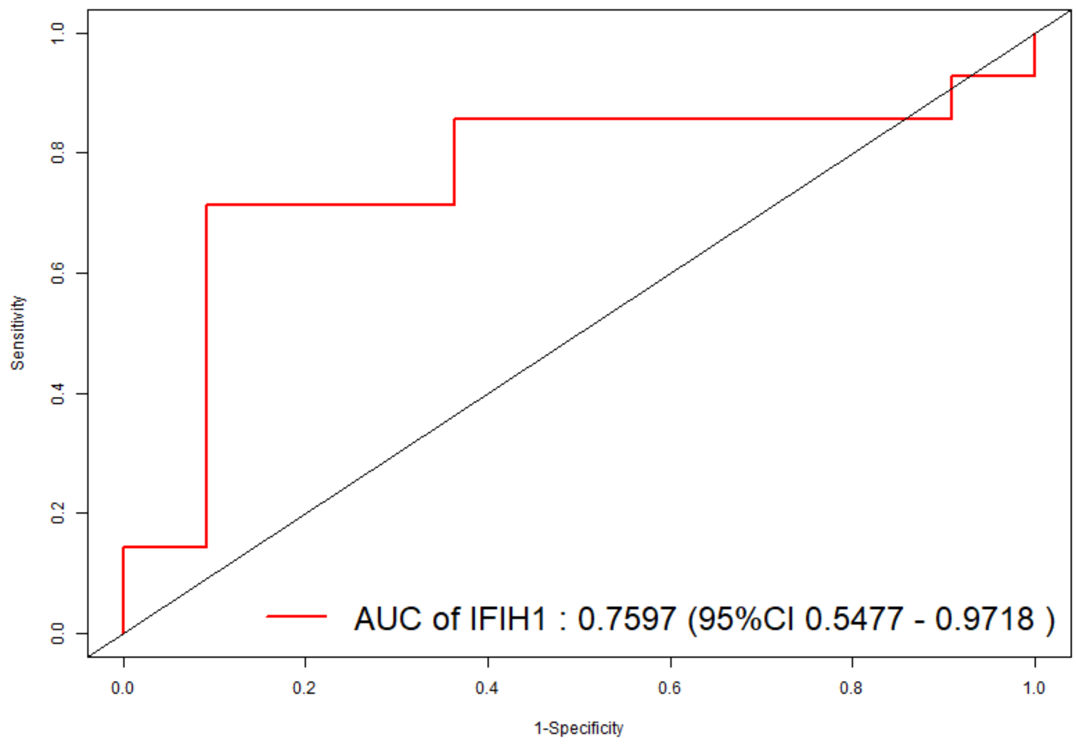

Supplement: Supplementary Data Sheet 1 — The ROC curves for each key target. [file DataSheet1.pdf]
