# Supplementary material for: Exploring the shared mechanism of fatigue between systemic lupus erythematosus and myalgic encephalomyelitis/chronic fatigue syndrome: monocytic dysregulation and drug repurposing
Source: Front Immunol. 2025 Jan 7;15:1440922. doi: 10.3389/fimmu.2024.1440922 (PMC11752880; doi:10.3389/fimmu.2024.1440922)

Suloctidil - IL1B

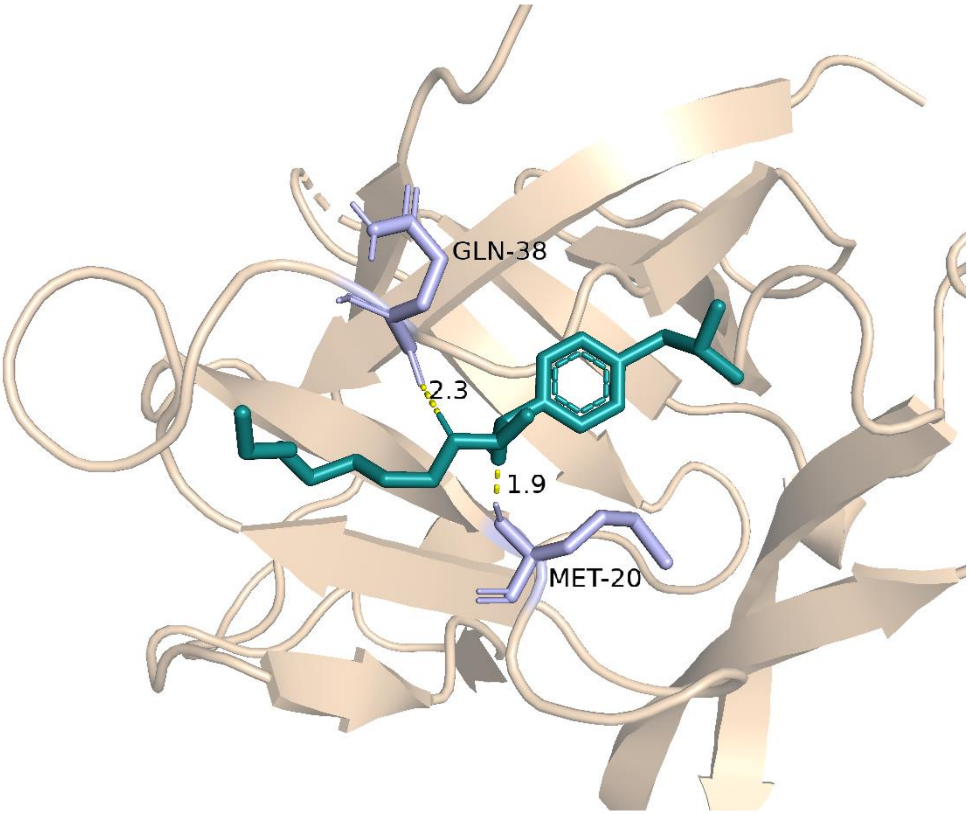

Suloctidil - CCL2

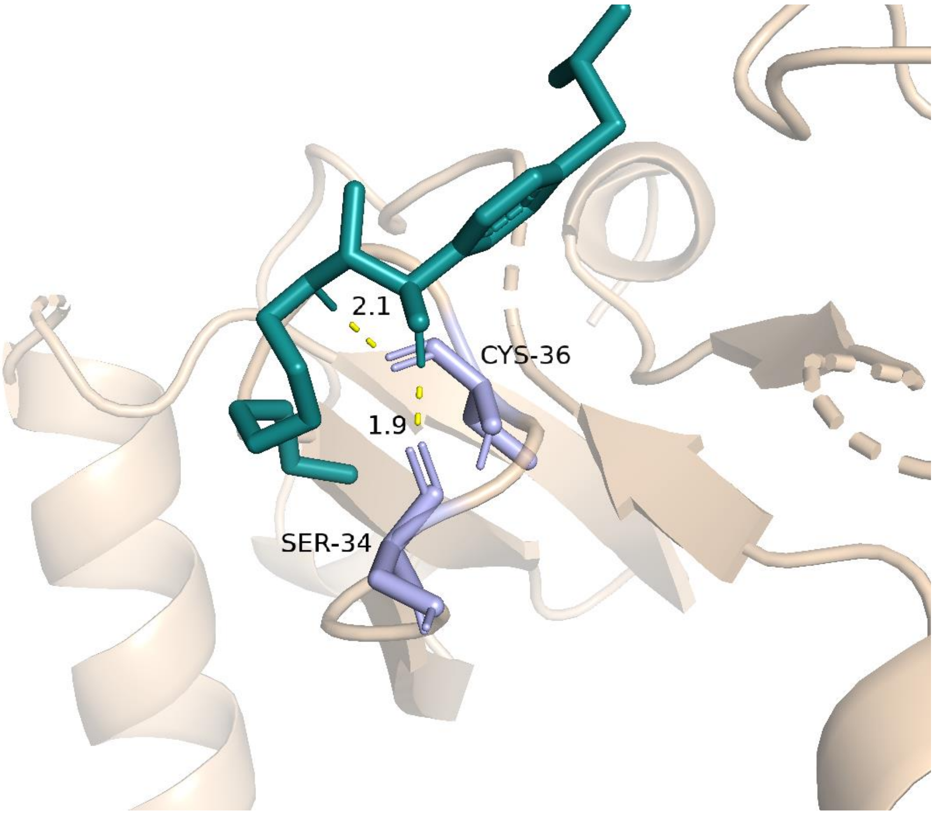

Suloctidil - TLR2

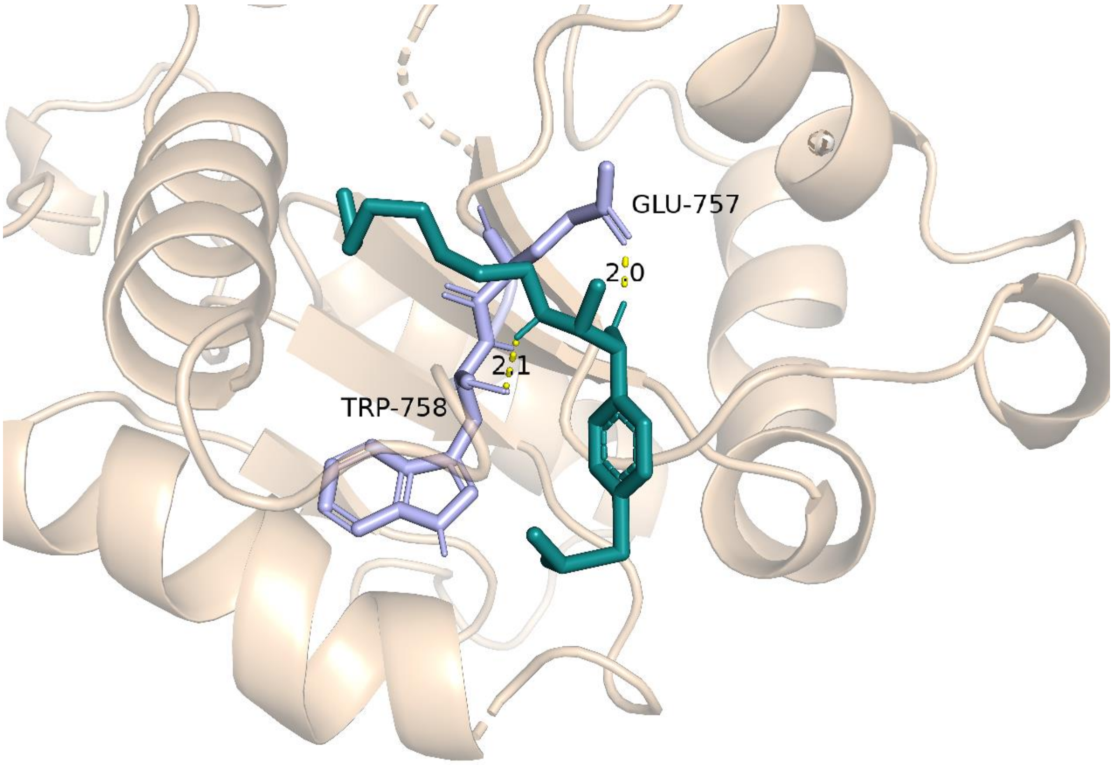

Suloctidil - STAT1

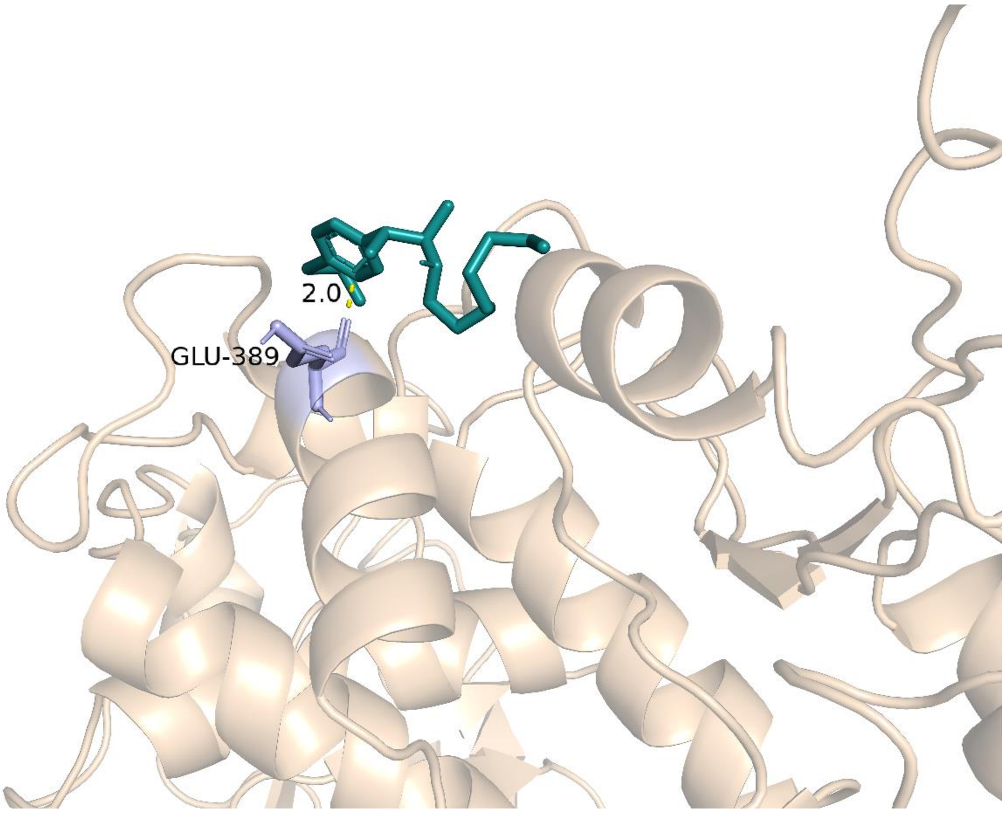

Suloctidil - IFIH1

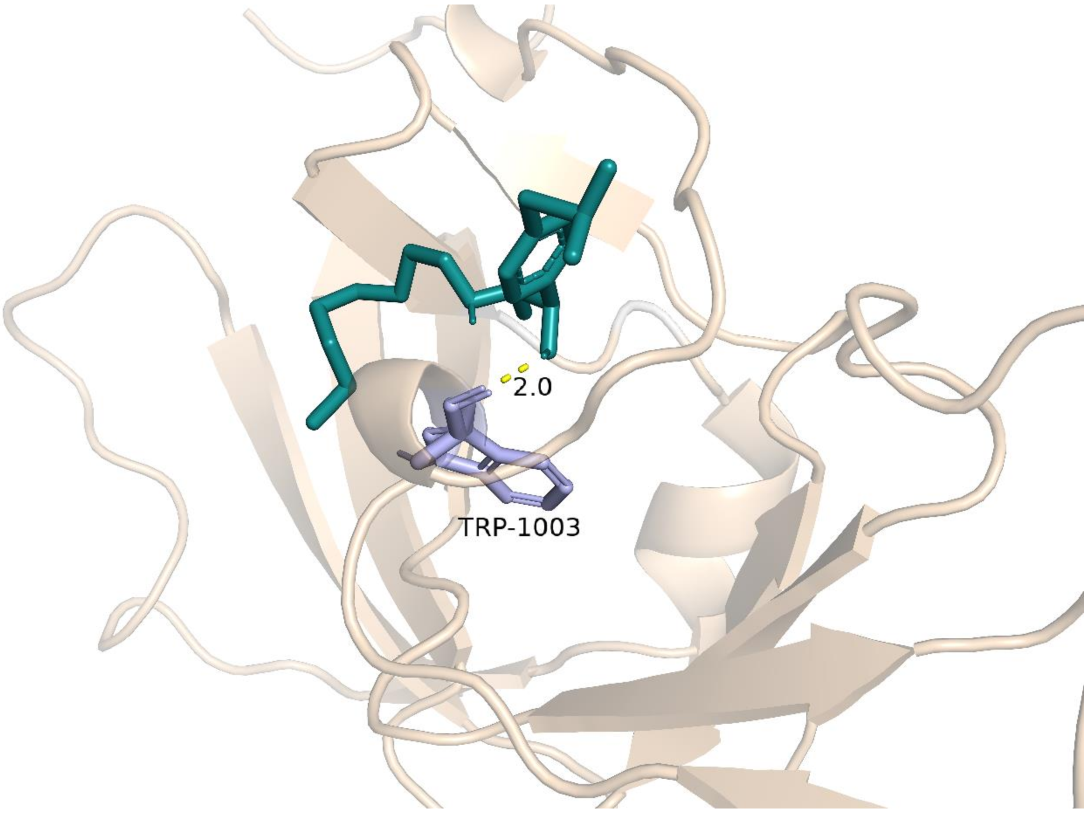

N-Acetyl-L-cysteine - IL1B

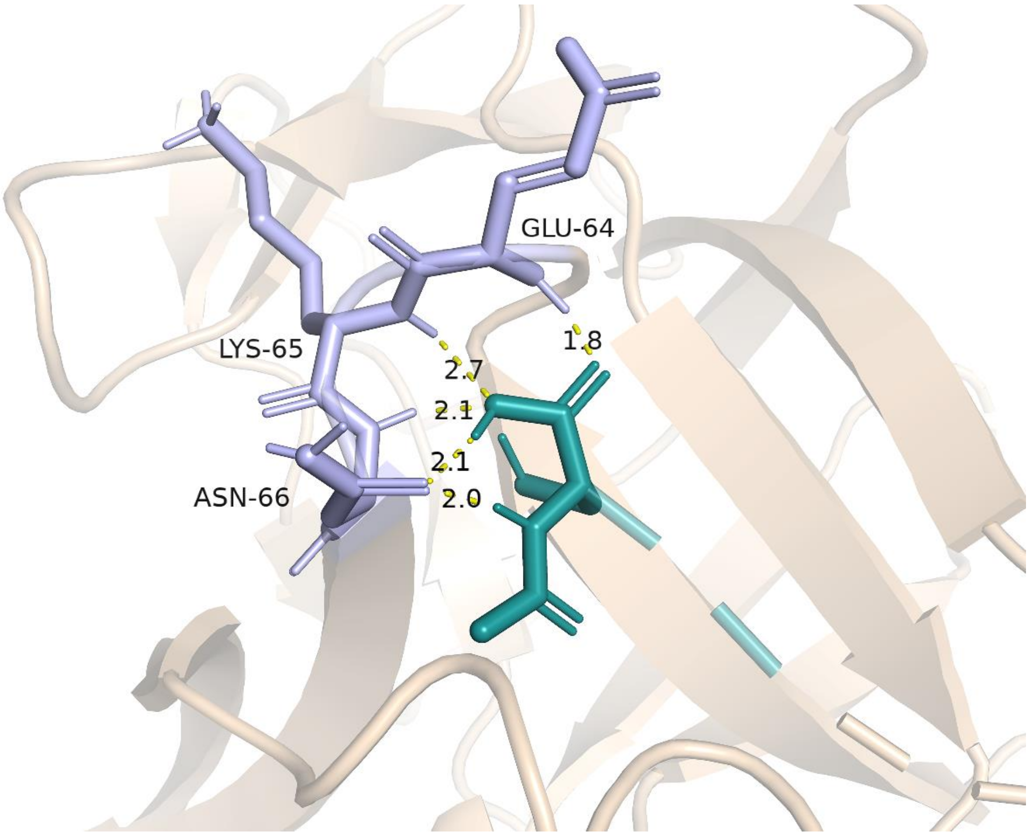

N-Acetyl-L-cysteine - CCL2

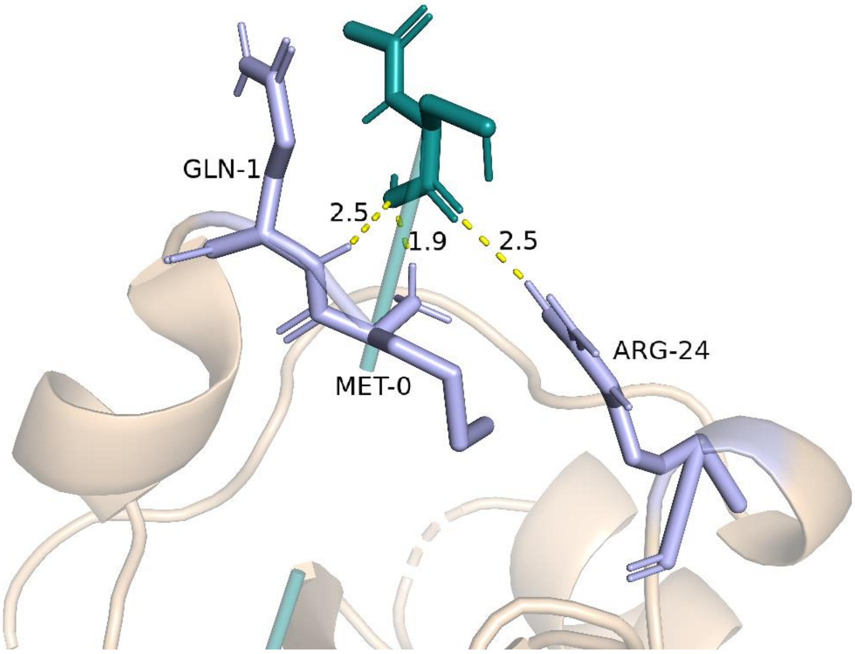

N-Acetyl-L-cysteine - TLR2

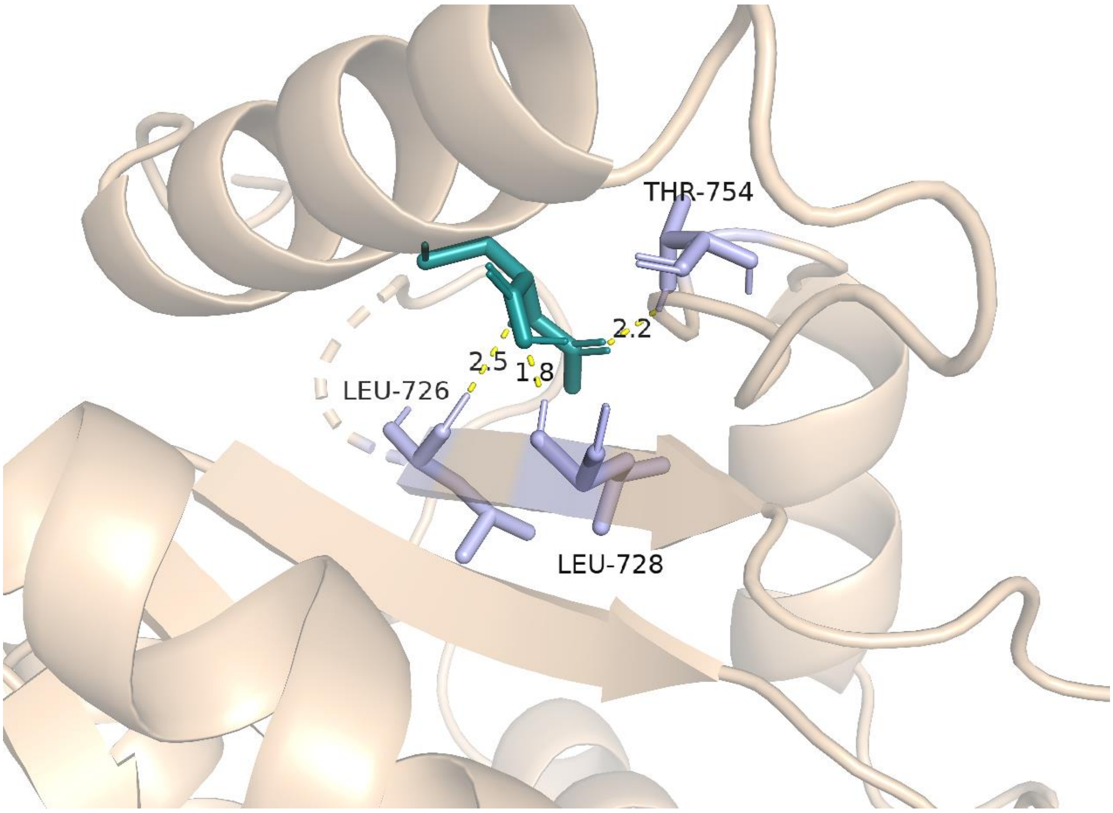

N-Acetyl-L-cysteine - STAT1

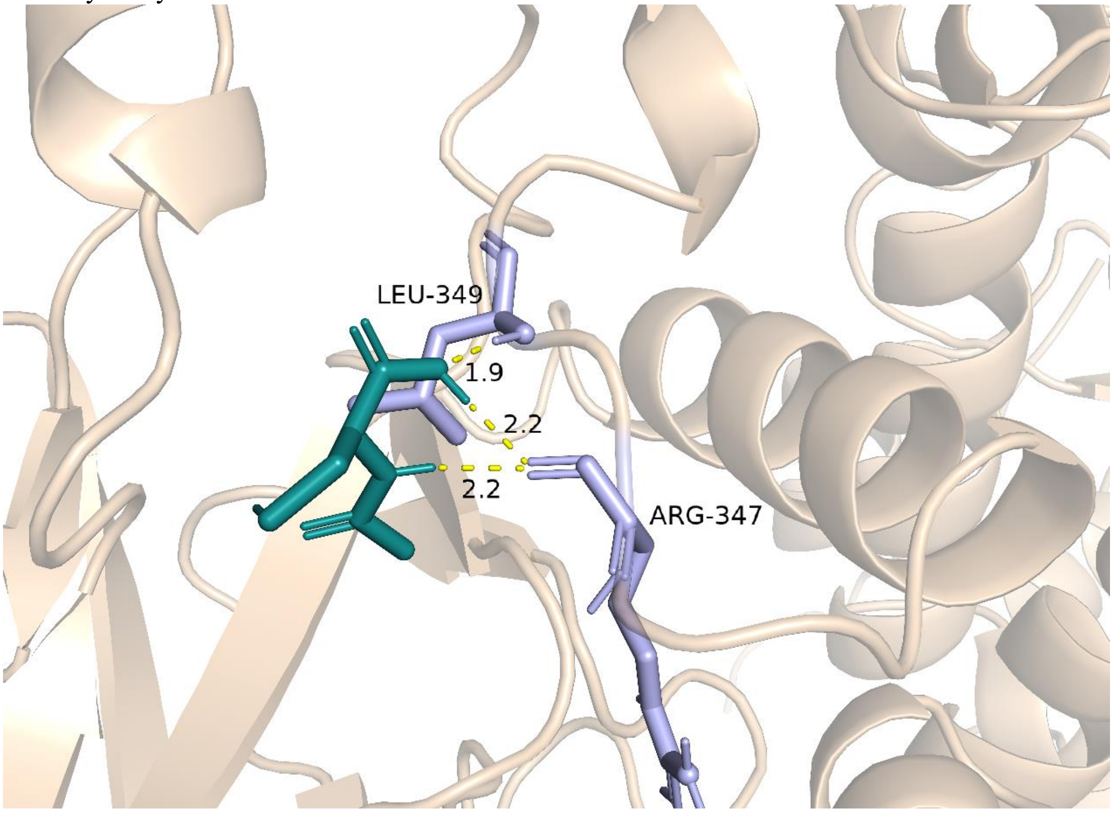

N-Acetyl-L-cysteine - IFIH1

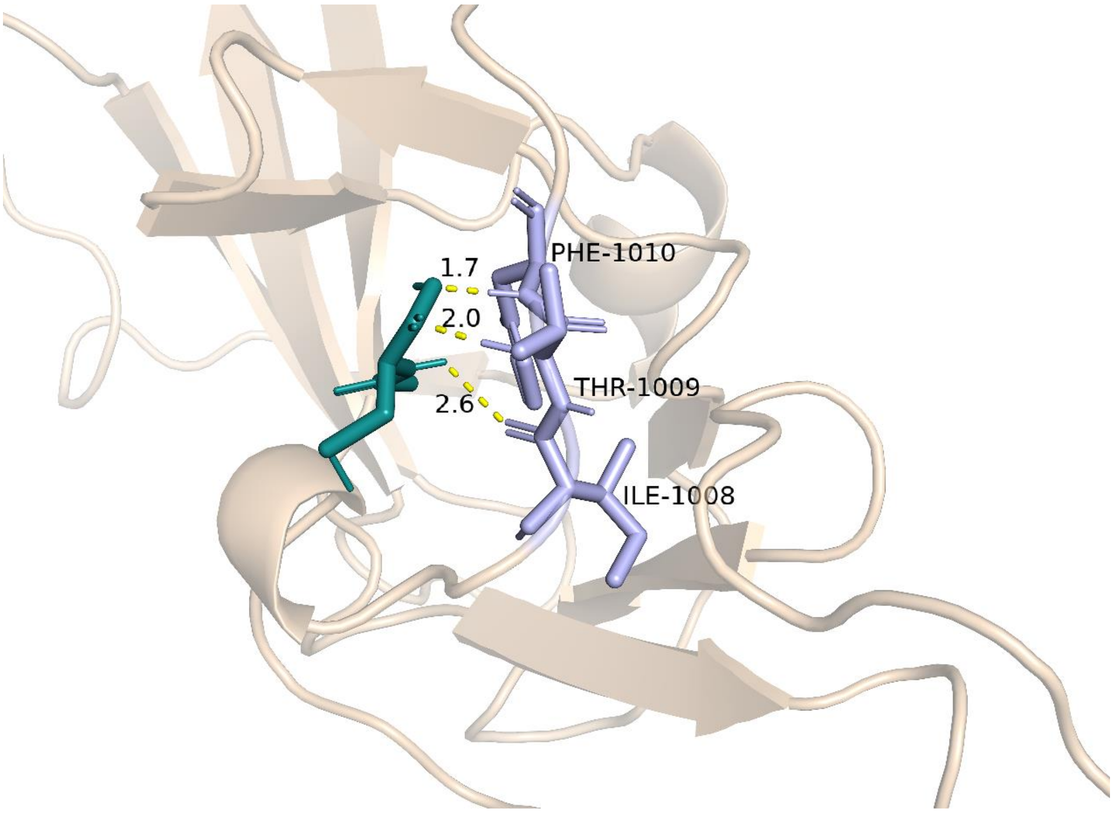

simvastatin - IL1B

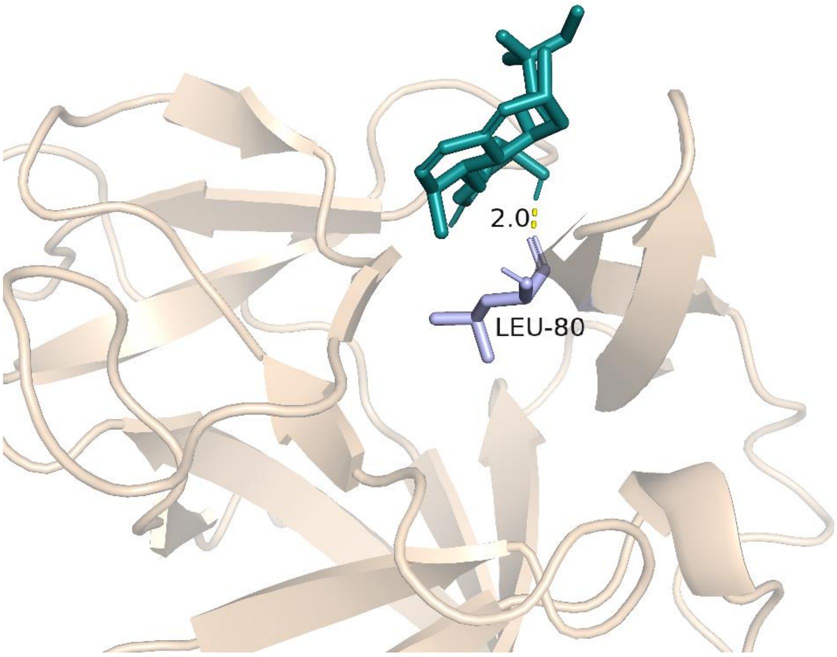

simvastatin - CCL2

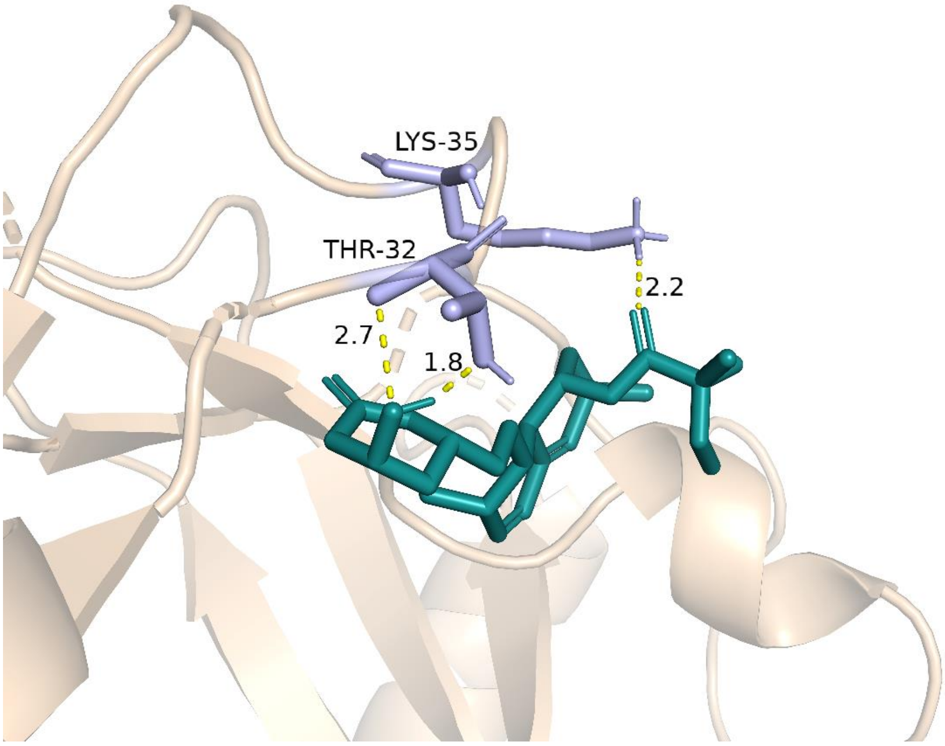

simvastatin - TLR2

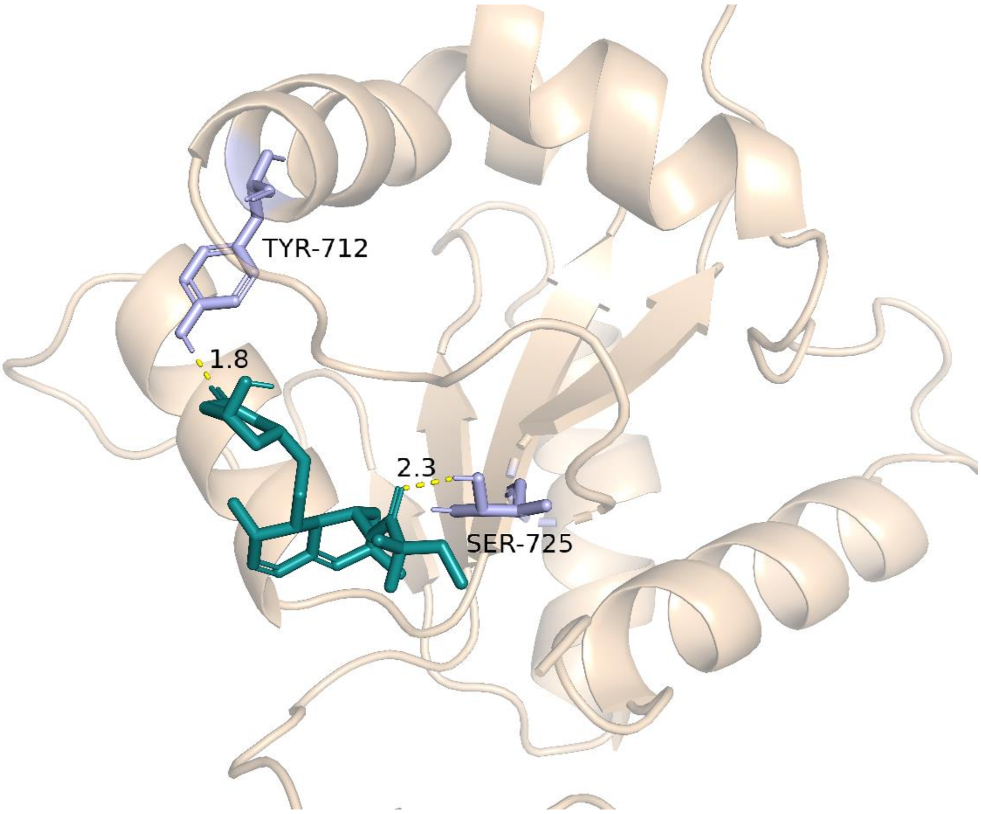

simvastatin - STAT1

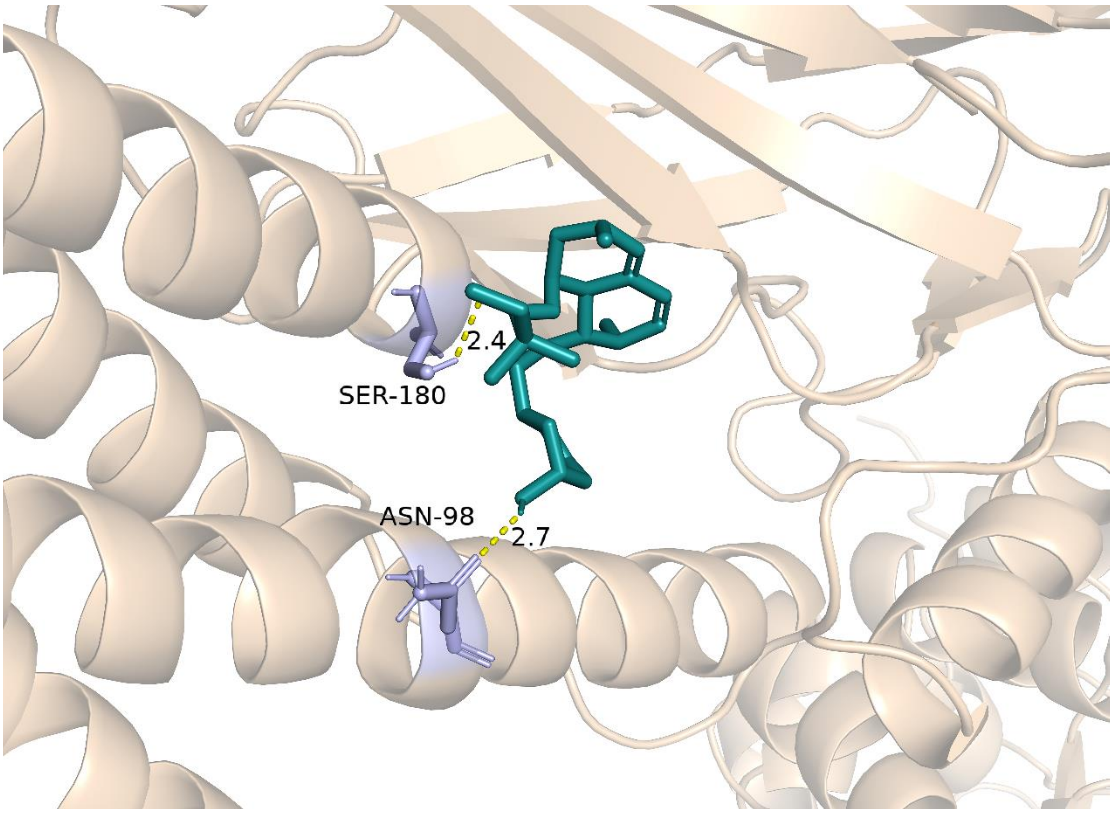

simvastatin - IFIH1

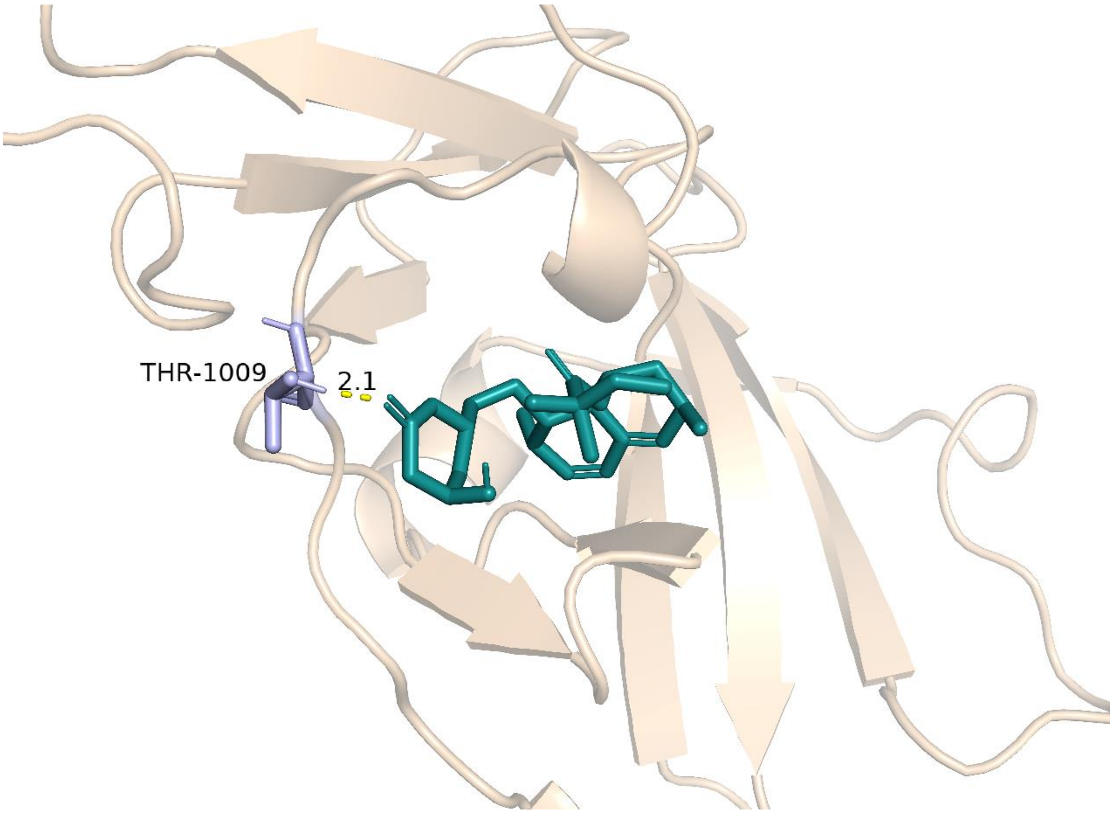

ACMC-20mvek - IL1B

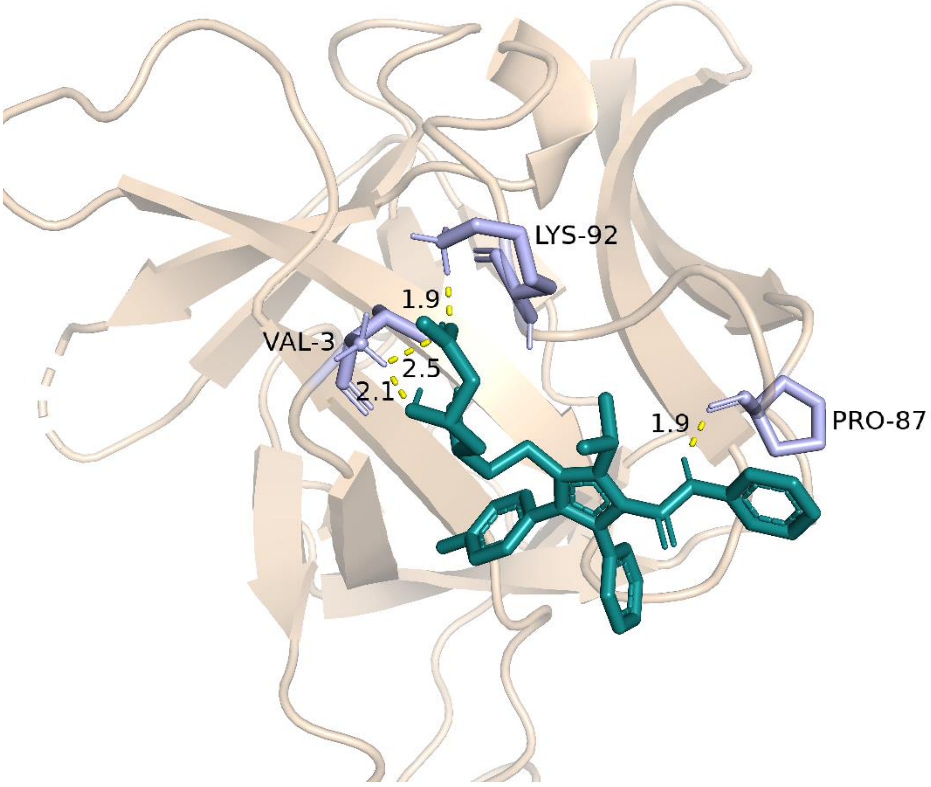

ACMC-20mvek - CCL2

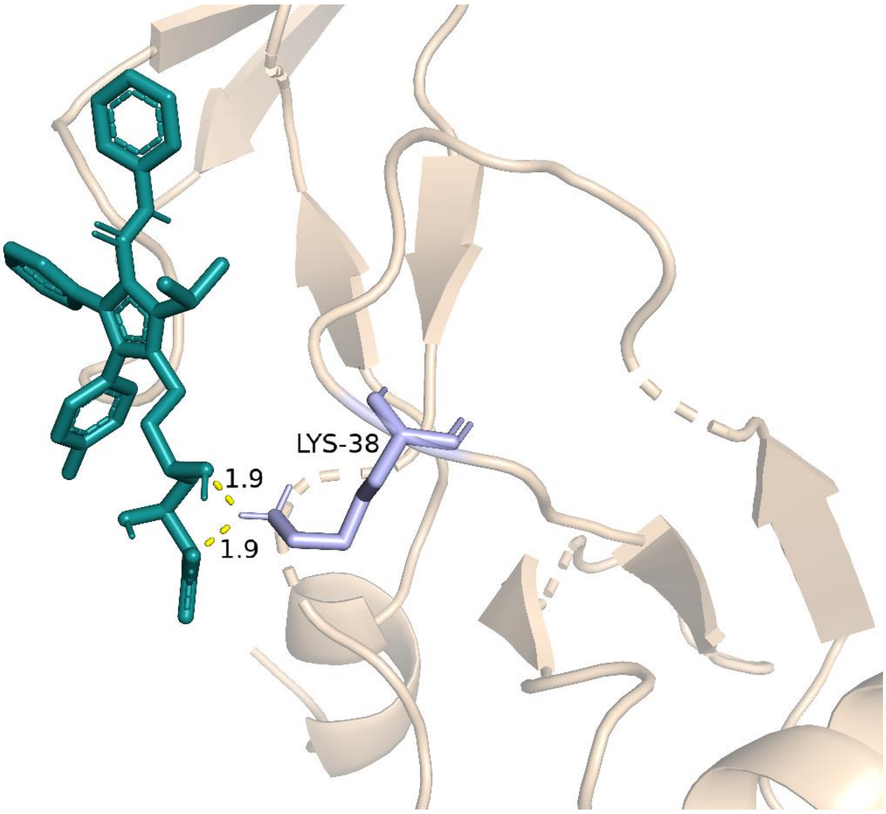

ACMC-20mvek - TLR2

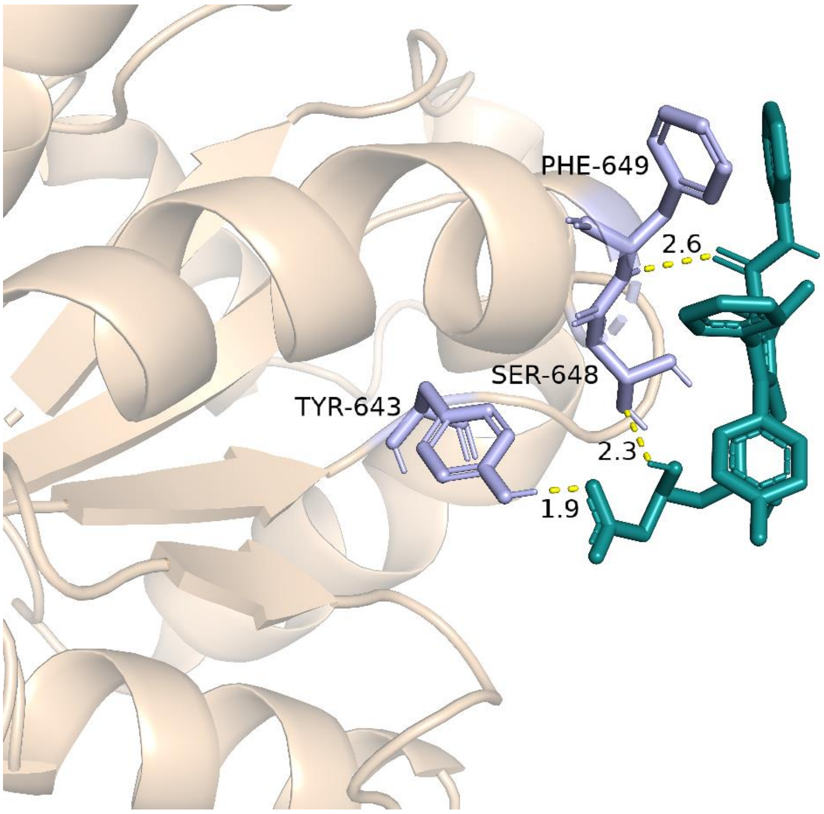

ACMC-20mvek - STAT1

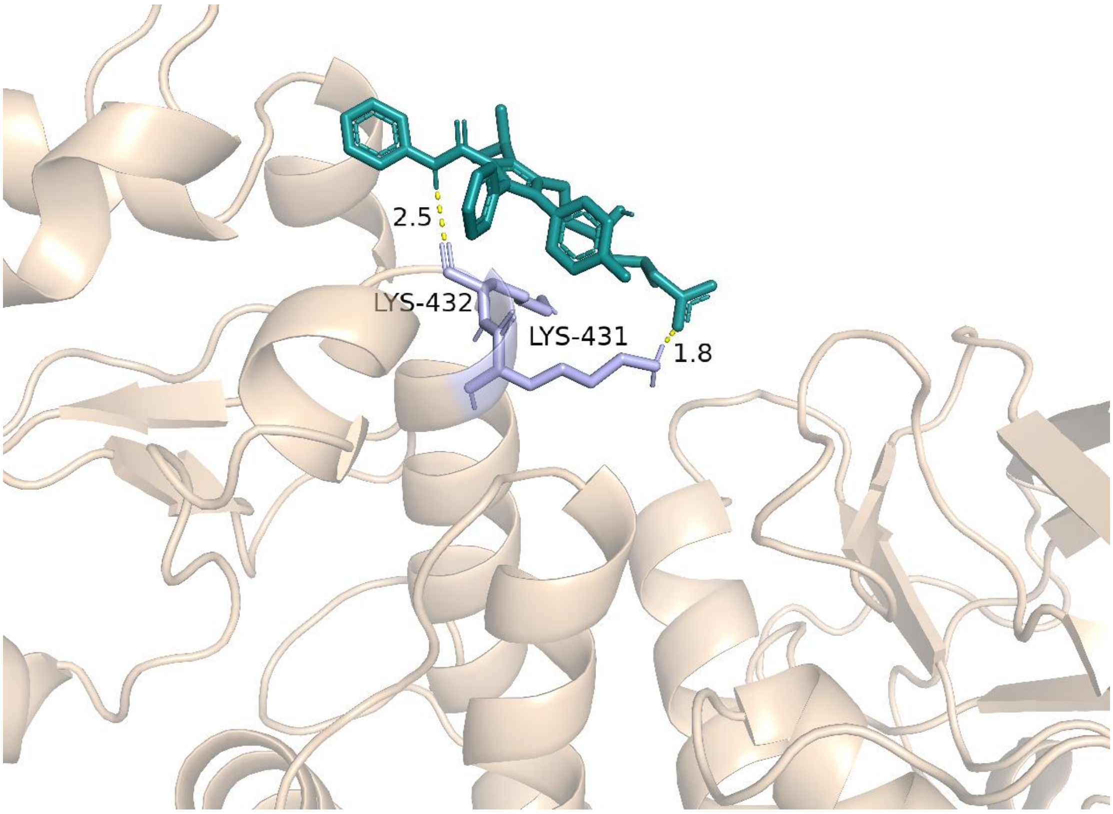

ACMC-20mvek - IFIH1

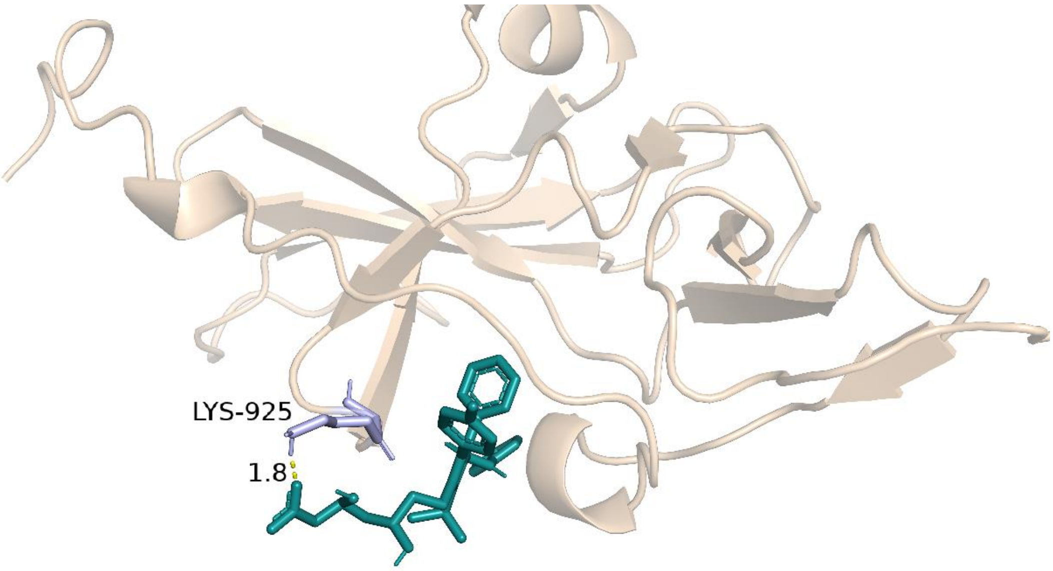

Camptothecin - IL1B

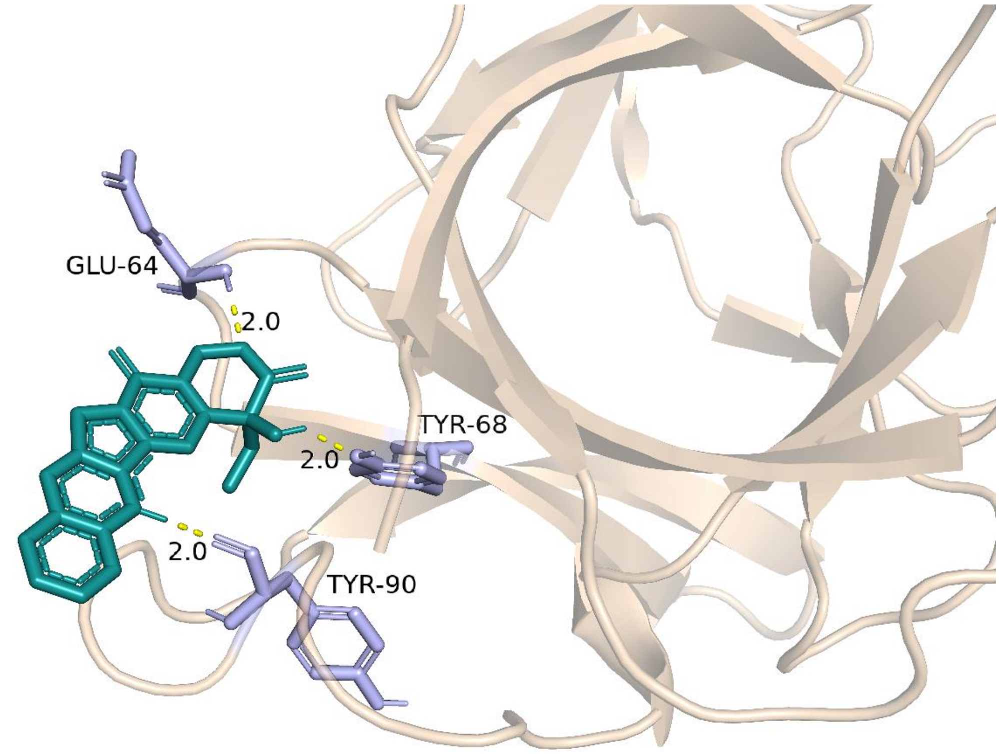

camptothecin - CCL2

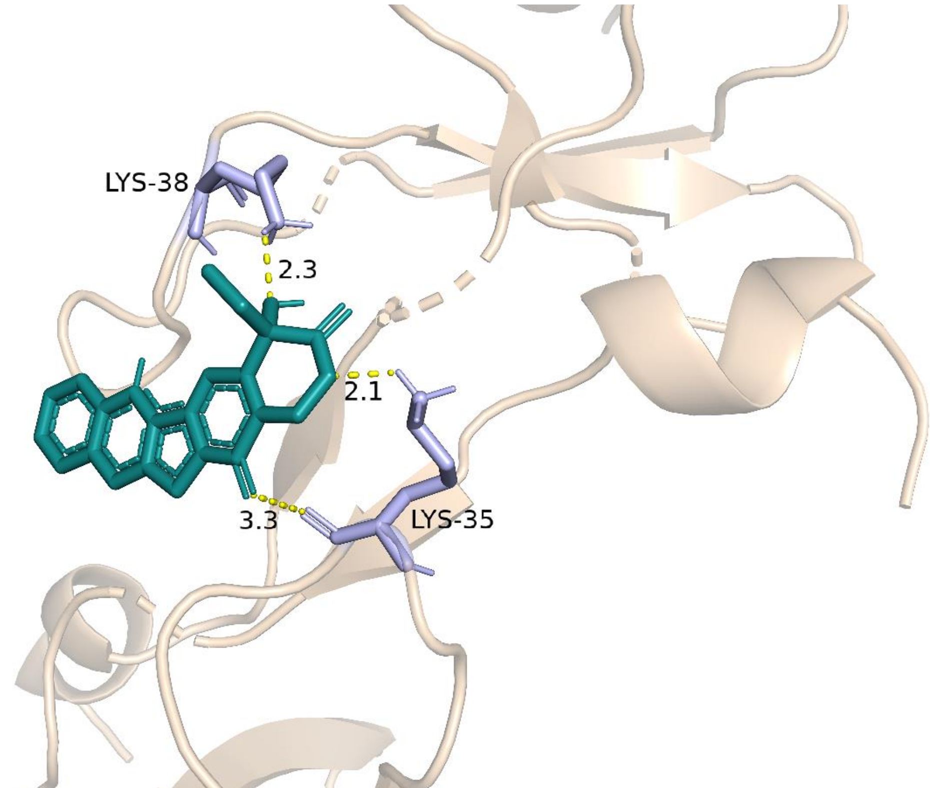

camptothecin - TLR2

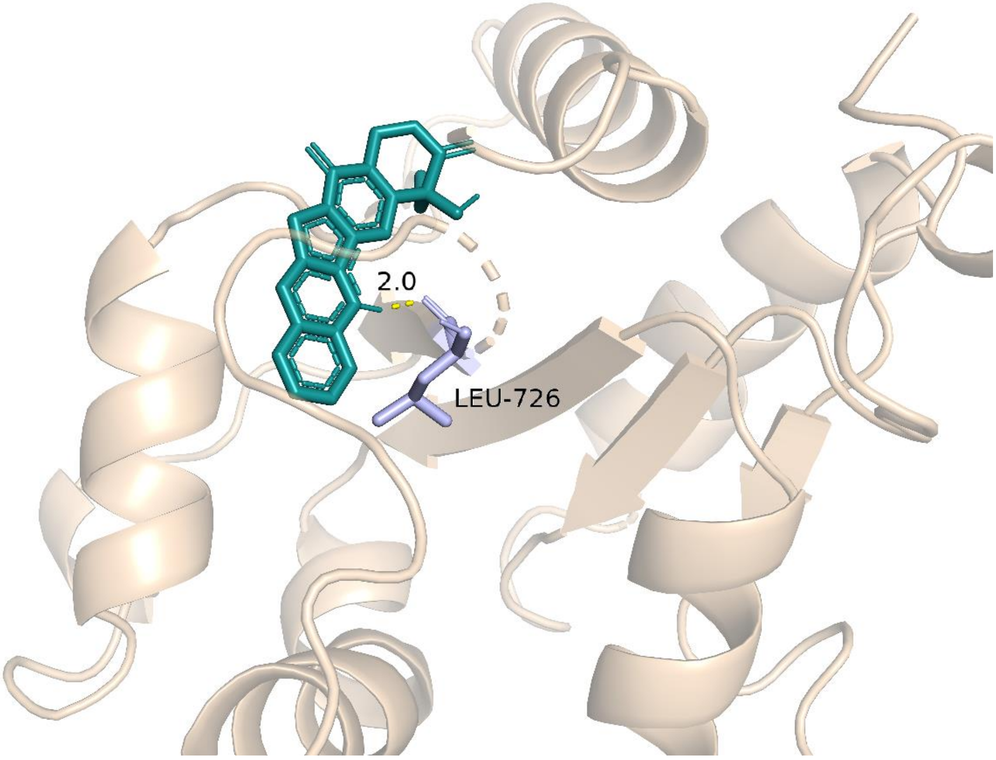

camptothecin - STAT1

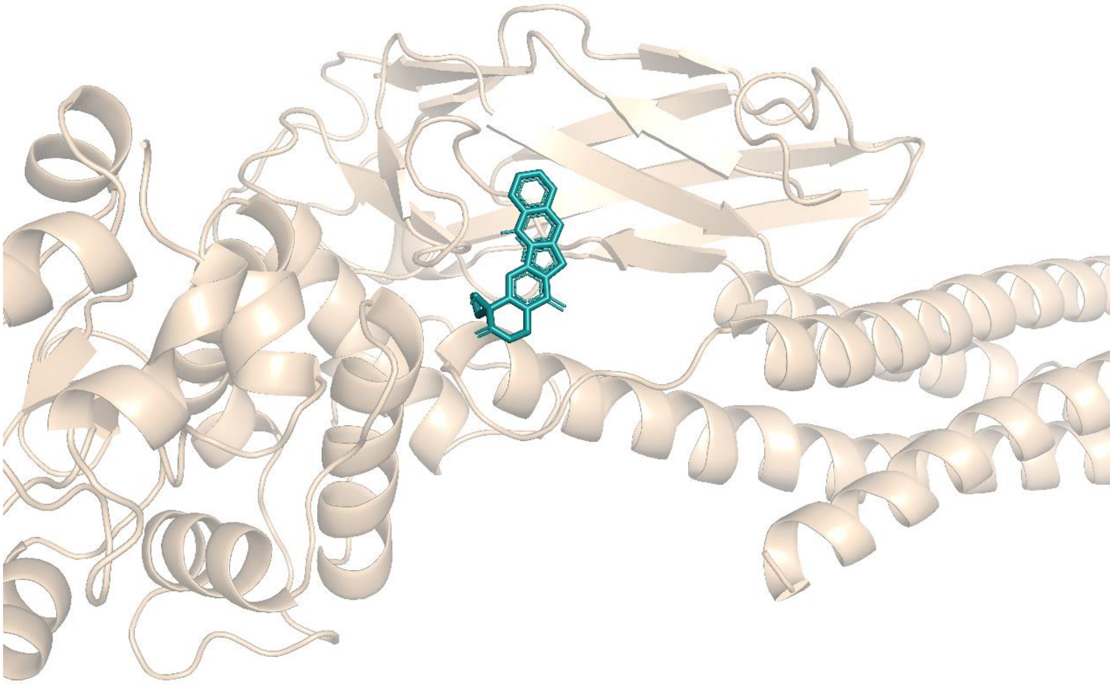

camptothecin - IFIH1

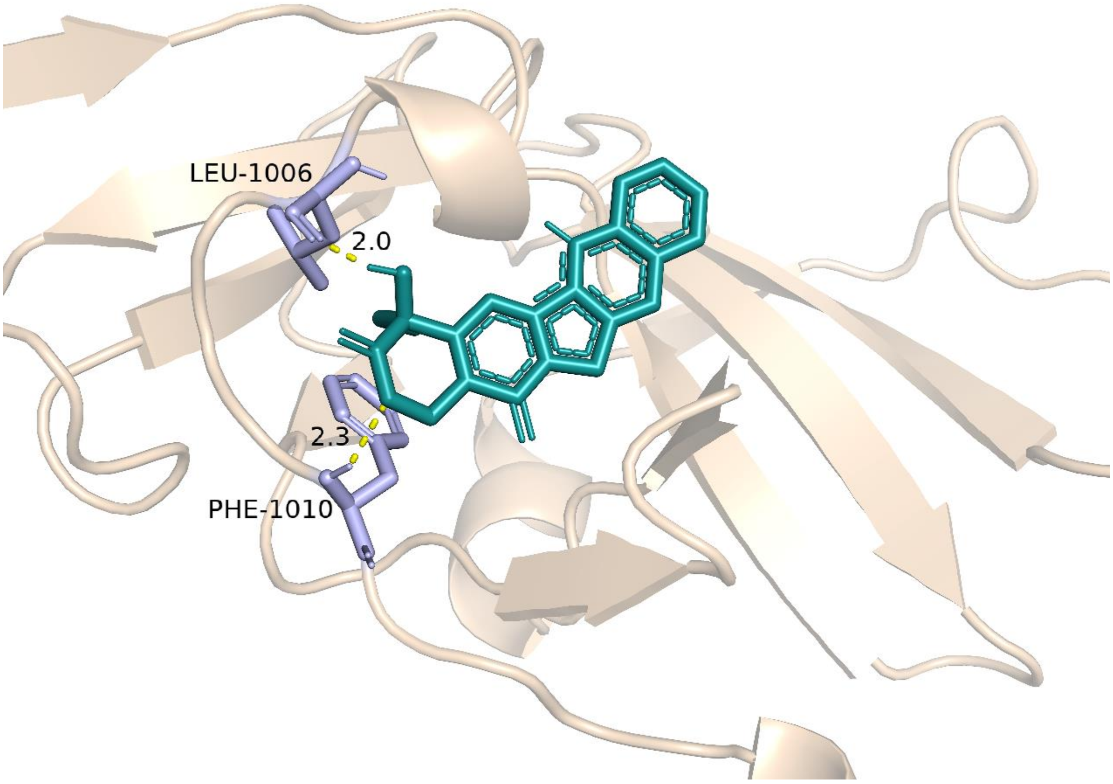

Supplement: Supplementary Data Sheet 2 — The molecular docking results. [file DataSheet2.pdf]
